# Analysis of genetic variability in Turner syndrome linked to long-term clinical features

**DOI:** 10.3389/fendo.2023.1227164

**Published:** 2023-09-20

**Authors:** Jenifer P. Suntharalingham, Miho Ishida, Antoinette Cameron-Pimblett, Sinead M. McGlacken-Byrne, Federica Buonocore, Ignacio del Valle, Gaganjit Kaur Madhan, Tony Brooks, Gerard S. Conway, John C. Achermann

**Affiliations:** ^1^ Genetics & Genomic Medicine Research and Teaching Department, UCL Great Ormond Street Institute of Child Health, University College London, London, United Kingdom; ^2^ Institute for Women’s Health, University College London, London, United Kingdom; ^3^ UCL Genomics, UCL Zayed Centre for Research into Rare Disease in Children, UCL Great Ormond Street Institute of Child Health, University College London, London, United Kingdom

**Keywords:** Turner syndrome, X chromosome, monosomy, diabetes mellitus, hypothyroidism, autoimmunity

## Abstract

**Background:**

Women with Turner syndrome (TS) (45,X and related karyotypes) have an increased prevalence of conditions such as diabetes mellitus, obesity, hypothyroidism, autoimmunity, hypertension, and congenital cardiovascular anomalies (CCA). Whilst the risk of developing these co-morbidities may be partly related to haploinsufficiency of key genes on the X chromosome, other mechanisms may be involved. Improving our understanding of underlying processes is important to develop personalized approaches to management.

**Objective:**

We investigated whether: 1) global genetic variability differs in women with TS, which might contribute to co-morbidities; 2) common variants in X genes - on the background of haploinsufficiency - are associated with phenotype (a “two-hit” hypothesis); 3) the previously reported association of autosomal *TIMP3* variants with CCA can be replicated.

**Methods:**

Whole exome sequencing was undertaken in leukocyte DNA from 134 adult women with TS and compared to 46,XX controls (n=23), 46,XX women with primary ovarian insufficiency (n=101), and 46,XY controls (n=11). 1) Variability in autosomal and X chromosome genes was analyzed for all individuals; 2) the relation between common X chromosome variants and the long-term phenotypes listed above was investigated in a subgroup of women with monosomy X; 3) *TIMP3* variance was investigated in relation to CCA.

**Results:**

Standard filtering identified 6,457,085 autosomal variants and 126,335 X chromosome variants for the entire cohort, whereas a somatic variant pipeline identified 16,223 autosomal and 477 X chromosome changes. 1) Overall exome variability of autosomal genes was similar in women with TS and control/comparison groups, whereas X chromosome variants were proportionate to the complement of X chromosome material; 2) when adjusted for multiple comparisons, no X chromosome gene/variants were strongly enriched in monosomy X women with key phenotypes compared to monosomy X women without these conditions, although several variants of interest emerged; 3) an association between *TIMP3* 22:32857305:C-T and CCA was found (CCA 13.6%; non-CCA 3.4%, p<0.02).

**Conclusions:**

Women with TS do not have an excess of genetic variability in exome analysis. No obvious X-chromosome variants driving phenotype were found, but several possible genes/variants of interest emerged. A reported association between autosomal *TIMP3* variance and congenital cardiac anomalies was replicated.

## 1 Introduction

Turner syndrome (TS) affects at least 1:2500 newborn females, where there is partial or complete loss of the second sex chromosome (1–6). The age of presentation in TS varies (2). Some girls with TS are diagnosed *in utero* or soon after birth due to congenital renal anomalies, heart anomalies (coarctation of aorta, bicuspid aortic valve) or lymphedema (2, 5, 6). Others are diagnosed later in childhood due to short stature, recurrent otitis media or congenital heart defects, or during teenage years due to primary amenorrhea or absent puberty (7). Prompt diagnosis and multidisciplinary support can allow for age-appropriate management including growth hormone therapy, pubertal induction and psychological input when needed (8, 9).

In adulthood, women with TS also have a higher prevalence of common conditions such as diabetes mellitus (DM), autoimmunity, hypothyroidism, hypertension, and cardiovascular disease (2, 4, 6, 8, 10). These features often contribute to excess mortality in women with TS when compared to the general population (4, 6, 10–12). Identifying specific genes or pathways contributing to the underlying mechanisms of these conditions is important for developing personalized approaches to treatment for women with TS and for targeting of long-term health surveillance.

The association between karyotype and phenotype in women with TS is still unclear. Karyotypes found in TS include aneuploidies (e.g. monosomy 45,X [40-50%], 45,X/46,XX mosaicism [15-25%], 45,X/46,XY [3-10%], 45,X/47,XXX [3%]), as well as structural X chromosome variants (isochromosome Xq (46,X,i(Xq) or 45,X/46X,i(Xq) mosaicism [10%], 45,X/46,X,r(X) ring mosaicism [7%], or rarer Xp and Xq deletions) (4, 8). Several clinical features have been reported to be more prevalent with specific karyotypes. For example, a 45,X karyotype may have the highest morbidity, whereas women with a 45,X/46,XX mosaic karyotype are less prone to obesity and hypertension and generally have the fewest comorbidities (2, 4). Autoimmune diseases, hearing loss and congenital cardiac features have sometimes been reported to be associated with an isochromosome karyotype, but studies are inconsistent (4, 13–17). Women with ring X karyotypes may have a predisposition towards elevated HbAc1 and alanine transaminase (ALT) (4), indicating a potential increased risk of diabetes and fatty liver disease. Tissue level mosaicism (45,X/46,XX, 45,X/46,XY) has also been proposed to influence phenotype, but strong data for this effect are limited (2, 3, 18).

The mechanism (or mechanisms) by which aneuploidy or structural variance of the X chromosome gives rise to the broad range of phenotypes in TS is still under investigation (2, 19–22).

**Figure 1:**
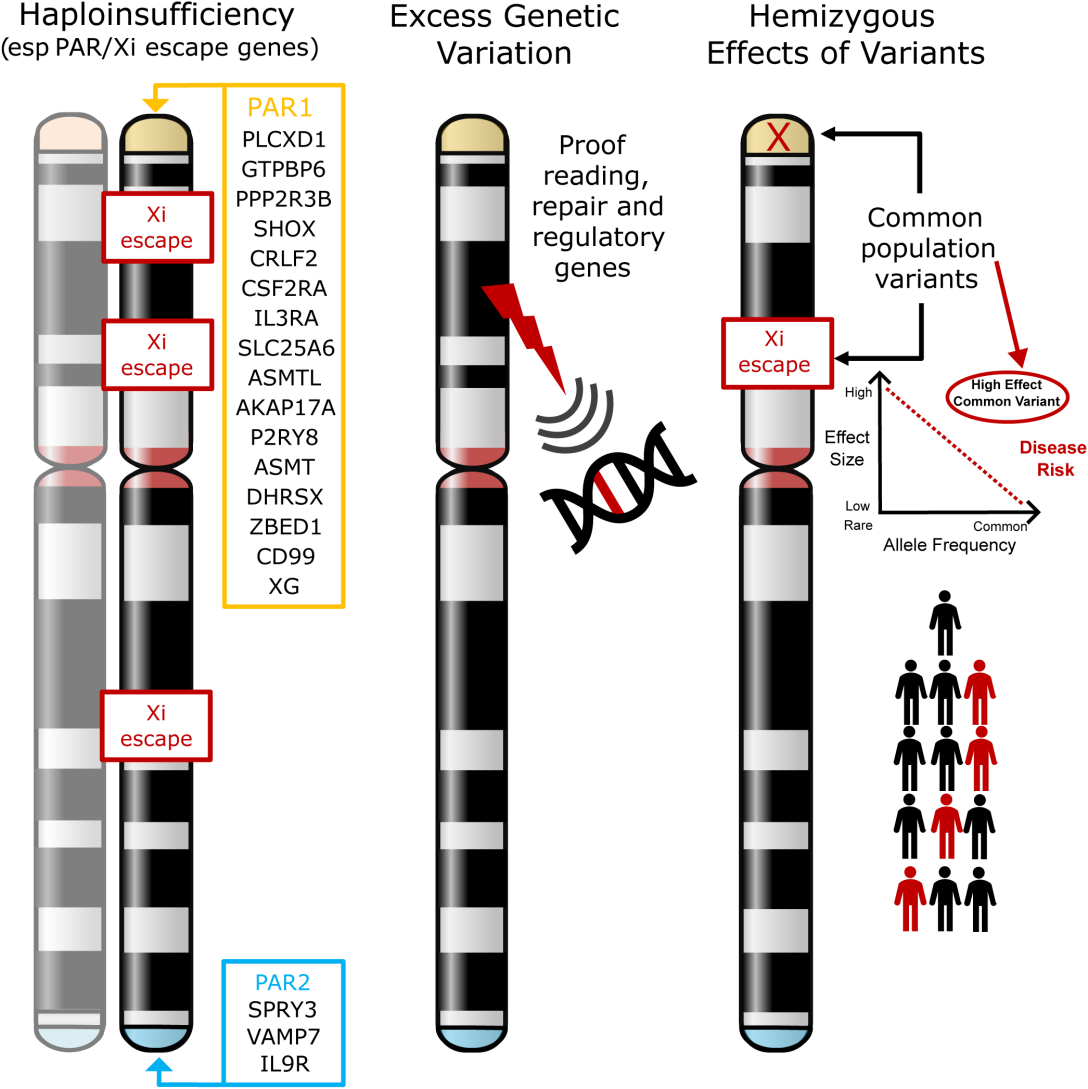
Models of potential mechanisms leading to phenotypes in Turner Syndrome. The most established theory involves haploinsufficiency of X chromosome genes (especially in the pseudoautosomal regions (PAR1, PAR2)) or in genes that escape X inactivation (Xi) (*left illustration*). The hypotheses tested in this current work are whether loss of X chromosome genes results in more widespread genomic variability (for example, through loss of a DNA repair or proof-reading gene) (*middle illustration*), or whether in the presence of haploinsufficiency, common hemizygous variants in genes (especially in the PAR region) can act as major modifiers of phenotype (*right illustration*). PAR, pseudoautosomal region; Xi, X inactivation.

The most established hypothesis is that TS phenotypes are largely a consequence of haploinsufficiency of genes that are normally biallelically expressed from both X chromosomes in women, and from the X and Y homologues in men. Most crucially this includes genes located in the pseudoautosomal regions (PAR), homologous regions present on the short arm (PAR1) and long arm (PAR2) of both sex chromosomes (X and Y) (2, 23, 24) (**Figure 1**, *left illustration*). PAR1 genes are typically haploinsufficient in all women with non-mosaic TS, irrespective of karyotype (20, 24). The clearest example of this is haploinsufficiency of the PAR gene *SHOX*, which is linked to short stature in TS (19, 25, 26). However, specific phenotypic effects of haploinsufficiency of other PAR genes is much less well established. Furthermore, haploinsufficiency of genes that escape X inactivation (Xi) may also be important. X chromosome inactivation is a mechanism whereby there is dosage compensation to prevent overexpression of X genes in diploid females. Up to 15% of genes escape X inactivation (21, 23, 27, 28), sometimes in a tissue- or time-specific manner, and are candidates for TS phenotypes as their net expression level would be reduced compared to when two X chromosomes (46,XX) are present.

In addition to haploinsufficiency of PAR gene or genes that escape X-inactivation, several other hypotheses exist for contributory mechanisms to phenotypes in women with TS. One hypothesis is that disruption of genes on the X chromosome has a “knock-on” or “ripple” effect elsewhere in the genome, either on the X chromosome itself or on autosomal loci (22). For example, haploinsufficiency of key genes may affect autosomal transcription (e.g., *ZFY*, *ZBED1*), translation (e.g., *DDX3X*, *EIF1AX*, *RPS4X*), splicing (e.g., *DDX3X*, *AKAP17A*) and DNA methylation/chromatin modification (e.g., *KDM5C*, *KDM6A*, *TBL1X*, *USP9X*), with ZFY having a potential key regulatory role in this regard (2, 22, 27, 29–32). Circular RNA and other global changes may also be implicated (31, 33, 34). Importantly, additional second variants (for example in autosomal genes) have also been proposed to contribute to phenotype when combined with haploinsufficiency of a PAR gene or a gene that may escape Xi. A reported example of this is where second variants in the autosomal gene *TIMP3* likely combine with disruption of the related X chromosome gene *TIMP1* to increase the risk of cardiovascular anomalies (e.g., bicuspid aortic value) in a “two-hit” mechanism (35, 36).

In this study, we aimed to expand on these concepts to address three hypotheses:

1) Whether disruption of an X chromosome gene with a proofreading or DNA repair function has a wider “ripple” effect on the genome, resulting in increased genetic variability that might contribute to excess morbidity and other features (**Figure 1**, *center illustration*).
2) Can common hemizygous variants in X chromosome genes (especially in the PAR region or in genes that escape X inactivation) contribute to the risk of associated features in TS (**Figure 1**, *right illustration*). Having a single copy (haploinsufficiency) of a gene in this region may already infer a risk for a given phenotype. We hypothesize that in this scenario, common population variants in the remaining expressed allele could have an unexpectedly strong influence on phenotype, as this is a unique biological situation where they are present in a hemizygous (monoallelic) state. Thus, the functional influence of common variants could be “exposed” and have quite a marked effect on the risk of developing conditions such as DM and autoimmunity (“X chromosome two-hit hypothesis”).
3) If variants in the autosomal gene *TIMP3*, when coupled with loss of *TIMP1* (X chromosome) are associated with an increased risk of congenital cardiac anomalies (“autosome two-hit hypothesis”), as has been reported previously (35, 36).

In order to investigate these hypotheses further, we undertook a large-scale genetic analysis in 134 women with monosomy X and associated karyotypes and investigated genetic variability in relation to control groups and phenotypic features.

## 2 Materials and methods

### 2.1 Cohorts and setting

#### 2.1.1 Turner syndrome

The study was conducted as part of the Reproductive Life Course Project (IRAS ID 184846; NRES Committee London-Chelsea (16/LO/0682)) at University College London Hospitals (UCLH), London, UK. A total of 134 women were recruited from the UCLH Turner Syndrome clinic (2015-2019) overseen by specialist multi-disciplinary professionals. All women provided written, informed consent to take part (median age 35.7 years; range 19.2 to 68 years).

The original diagnosis was made by G-banded karyotype analysis undertaken by routine clinical cytogenetic services. A mosaic screen of at least 30 cultured lymphocytes was typically done. Following recruitment to this study, single nucleotide polymorphism (SNP) array analysis was undertaken on a recent leukocyte-derived DNA sample (see below). Women were excluded if they had evidence of 46,XX or 46,XY mosaicism, or if a Y fragment was identified on original karyotype or on recent SNP array analysis. Women with complex structural variance or complex aneuploidies with a 46,XX or 47,XXX cell line were also excluded.

Within this cohort, three women who were originally reported to have a non-mosaic 46,X,i(Xq) karyotype were subsequently found to have a low-level mosaic (45,X) line on SNP analysis (45,X/46,X,i(Xq)). Three other women had karyotypes that were discordant with the original results, but in these cases historical records were limited. No women had a significant Y line or 46,XX line present. In all situations the most recent SNP array karyotype was used for grouping in this study (**Table 1**).

**Table 1:** Overview of cohorts studied and related karyotypes.

Cohort	Karyotype	n
Turner syndrome: “Monosomy”	45,X	75
Turner syndrome: “Ring”	45,X/46,X,r(X)	20
Turner syndrome: “Complex”	46,X,del(Xp)/Complex	5
Turner syndrome: “Isochromosome”	46,X,i(Xq) and variants	34
46,XX: Control	46,XX	23
46,XX: Primary Ovarian Insufficiency	46,XX	101
46,XY: Control	46,XY	11

“Isochromosome” used here and elsewhere to include isodicentric Xq (i(Xq)). n, number.

Key clinical parameters that were chosen for further analysis in relation to genetic variability were DM/impaired glucose tolerance (IGT), obesity, autoimmune disease, hypothyroidism, hypertension, congenital cardiovascular anomaly, and hearing loss. These conditions were defined as:

1) Diabetes mellitus: A fasting plasma glucose of ≥7.0 mmol/l (126 mg/dL), or ≥11.1 mmol/l (200 mg/dL) after 120 minutes on a standard oral glucose tolerance test (75g oral glucose). Impaired glucose tolerance: A plasma glucose of between 7.8 mmol/l (140 mg/dL) and 11.1 mmol/l (200 mg/dL) after 120 minutes on a standard oral glucose tolerance test (75g oral glucose). For the purposes of analysis, we combined individuals with diabetes and individuals who had IGT under the label “Diabetes”.
2) Obesity: A body mass index (BMI) greater than 30 kg/m^2^.
3) Autoimmunity: A diagnosis of autoimmune disorders such as celiac disease, inflammatory bowel disease, or antibody positive hypothyroidism.
4) Hypothyroidism: An elevated TSH and long-term treatment with thyroxine replacement. The size of this subgroup allowed for this to be analyzed as a separate entity and may have included women with autoimmune hypothyroidism who were no longer auto-antibody positive.
5) Hypertension: A persistent elevation in blood pressure (140/90 mmHg or higher) and treated with long-term antihypertensive therapy.
6) Congenital Cardiovascular Anomaly (CCA): The presence of a bicuspid aortic valve or coarctation of the aorta or any form of cardiac surgery in childhood. Unfortunately, serial aortic root dimension data in adulthood were not available for analysis.
7) Hearing loss: The use or recommended use of a hearing aid.

#### 2.1.2 Comparison group: primary ovarian insufficiency

For comparisons of global genetic (exome) variability, data from 101 women with POI were obtained from the Reproductive Life Course Study at University College London Hospitals, London. Those with a known cause of ovarian dysfunction (e.g., abnormal karyotype, iatrogenic POI) were excluded (37). Women recruited to this study provided written informed consent for genetic analysis as part of the Reproductive Life Course Study at University College London Hospitals (ethical approval: NRES Committee London-Chelsea [16LO0682]).

#### 2.1.3 Control group: human random controls

Control DNA samples (46,XX, n=23; 46,XY, n=11) were obtained from Human Random Control DNA Panels (European Collection of Cell Cultures, Public Health England, Sigma-Aldrich).

### 2.2 DNA extraction from whole blood

Total DNA was extracted and purified from whole blood using a QIAamp Blood Maxi Kit or QIAamp Blood Midi Kit (Qiagen, Hilden, Germany), following the manufacturer’s protocol. Samples were submitted for SNP array and exome sequencing through UCL Genomics.

### 2.3 Genotyping and mosaicism analysis

SNP array analysis was undertaken using Illumina Global Screening Arrays (v3.0) containing 654,027 markers, following the Infinium HTS Assay Reference Guide (# 15045738 v04) (Illumina, Inc. San Diego, CA, USA). Raw data files were analyzed in Illumina Genome Studio version 2.0 and X chromosome mosaicism calculations were made using methods described by Conlin et al. (38).

### 2.4 Custom exome sequencing

#### 2.4.1 Exome capture and sequencing

Exome sequencing was performed using a customized Nonacus Exome CG panel (Nonacus, Birmingham, UK) and Nonacus protocol (Protocol Guide v1.2.2) with minor modifications. In brief, 200ng of genomic DNA was used for exome pre-capture library preparation. Library preparations were carried out on a Hamilton StarLet robotic platform (Hamilton Company, Reno, NV, USA) and library qualitative checks were undertaken using Tapestation 4200 (Agilent Technologies, Santa Clara, CA, USA). Sample libraries were sequenced on a NovaSeq6000 Platform using an S4 flow cell (Illumina).

#### 2.4.2 Exome analysis and variant calling

FASTQ files were generated and processed through the bioinformatics pipeline shown in **Supplementary Figure 1**. Scripts for genome alignments and variant calling were provided by Nonacus. In brief, reads were aligned to the GRCh38 reference sequence using Burrows-Wheeler Aligner (BWA) and grouped by unique molecular identifiers (UMIs) with fgbio (v.0.4). Variant calling was undertaken with Platypus software (v0.8.1) with a default parameter, and multiple nucleotide polymorphisms (MNPs) were split with vcflib (v1.0.0).

**Figure 2:**
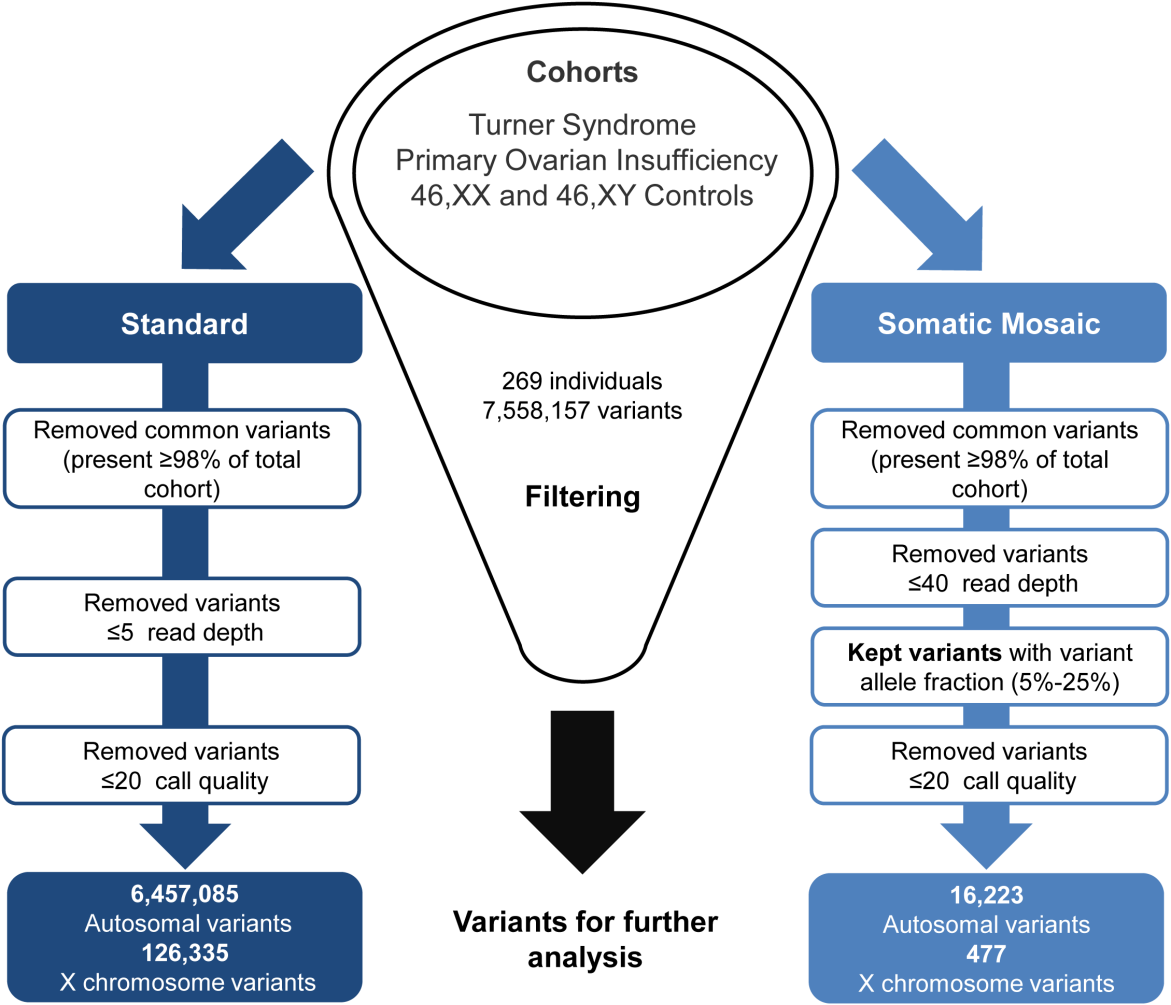
Exome data filtering analysis. The two parallel filtering approaches used for standard variant and somatic mosaic variant analysis.

Generated VCF files were uploaded to the Qiagen Clinical Insight (QCI) Interpret web-based platform for variant annotation and classification. All exome variants for all samples were exported from QCI as CSV files, these files were then merged and underwent two parallel approaches for standard variant and somatic mosaic variant filtering (**Figure 2**). For the somatic mosaic analysis, variants were included with at least 40X coverage and with a variant allele fraction of between 0.05 (5%) and 0.25 (25%). This approach was adopted to have enough sensitivity to detect genuine changes, whilst having high enough specificity to avoid “noise” at the lower range and potential heterozygous variants at the upper range. Analysis was undertaken using R version 4.1.1 (39). Variants were subcategorized according to predicted translational impact such as missense, synonymous, frameshift, and stop-gain. This approach provided a quantitative overview of the variety and types of variants in the TS cohort compared to the control/comparison groups described above.

#### 2.4.3 X chromosome variant enrichment analysis and phenotype

To investigate whether genetic variants in X chromosome genes (and especially PAR genes) are associated with phenotype, X chromosome variants from the standard filtering pipeline (**Figure 2**) were further analyzed in subgroups of women with monosomy X with and without the following conditions: DM/IGT (25 with versus 24 without), obesity (19 with versus 53 without), autoimmunity (24 with versus 28 without), hypothyroidism (32 with versus 43 without), hypertension (16 with versus 36 without), and CCA (for bicuspid aortic valve or coarctation of the aorta) (17 with versus 35 without). Analysis first involved filtering for common variants with the Genome Aggregation Database (gnomAD) allele frequency between 0.1 to 0.9 and quantifying the proportion of the “alternative” allele in the two monosomy X subgroups (condition versus no condition) at gene level (the number of unique individuals having a variant in each gene) and at variant level (the number of individuals with any given variant). The range 0.1 to 0.9 was used as we hypothesized that common population variants could influence phenotype, and that the proportion of these in condition versus non-condition groups would have to vary around a common population allele frequency. These cut-offs were used to accommodate a normally distributed effect size of 0.35 around a mean population value (see below), which would not be possible at extreme ends of allele frequency. Only the group of TS women with monosomy X were included in the initial study as the presence of a single X chromosome simplified analysis as only hemizygous variants had to be considered. Population control data for any variants of interest were obtained from gnomAD (v3.1.2) (accessed March 2023) (40). Only 46,XY control data were included as the presence of a single X chromosome simplified analysis because only hemizygous variants occur (rather than heterozygous or homozygous combinations). Fisher’s exact test and multiple comparison testing using the Bonferroni method was performed in R (39) to assess the significance of any findings. Genes and variants of interest were determined using an “effect size” greater than +0.35 or -0.35 between groups (i.e. the difference in alternative allele frequency in those with a condition compared to those without a condition). This effect size was calculated based on initial power calculations, and confirmed in a *post-hoc* power analysis based on final group size and ratios of those with and without a condition (**Supplementary Table 1**). Clinical conditions linked to these genes were determined using the Online Mendelian Inheritance in Man (OMIM) database (https://www.omim.org). Gene expression data for genes of interest were obtained using the consensus summary in the Human Protein Atlas (https://www.proteinatlas.org).

Following the initial analysis of X chromosome variants linked to phenotype in the 45,X monosomy subgroup, a separate analysis of PAR gene variance was undertaken in a wider cohort of women with additional TS-associated karyotypes using a similar design, as PAR1 genes are predicted to be haploinsufficient in all these women.

Analysis of “hearing loss” was also undertaken using the same pipelines (14 with versus 33 without), but these data are presented separately as the groups are smaller and the end points potentially less reliable.

### 2.5 *TIMP3* variants and congenital cardiovascular anomalies

In order to evaluate the link between *TIMP3* (chromosome 22) variants and CCA, exome sequencing data for variants in this gene were analyzed in relation to cardiac status in 95 women where phenotypic data were available. The minor allele frequency (MAF) of common variants detected was calculated and compared between groups (CCA [n=22] versus non-CCA [n=73]) using Fisher’s Exact testing. MAF was also compared to data obtained from gnomAD (v3.1.2) as well as previous reports of *TIMP3* variants in TS cardiac cohorts (35, 36).

### 2.6 Statistical analyses

#### 2.6.1 Mosaicism proportion

Where data were available, statistical analysis between the original and new percentage mosaicism values was undertaken in GraphPad Prism (version 9.5.5 for Windows, GraphPad Software, San Diego, CA, USA, www.graphpad.com) using the Wilcoxon matched-pairs signed rank test.

#### 2.6.2 Genetic variability and X chromosome variant enrichment analysis

Statistical analysis for genomic variability between TS and control/comparison cohorts was performed using a one-way ANOVA (Kruskal-Wallis) test in GraphPad Prism. For X chromosome variant filtering, and gene and variant level counts, Fisher and Bonferroni adjustment tests were undertaken in R using the tidyverse packages (39, 41). Bonferroni multiple adjustment comparisons were made using a very stringent approach taking into account all genes with data on the X chromosome. Graphical outputs were generated either using GraphPad Prism or using ggplot2 in R (41).

#### 2.6.3 TIMP3 variant enrichment analysis

Statistical analysis for potential enrichments of variants in *TIMP3* related to CCA was performed using Fisher’s Exact tests in GraphPad Prism.

## 3 Results

### 3.1 Overview of cohort

**Figure 3:**
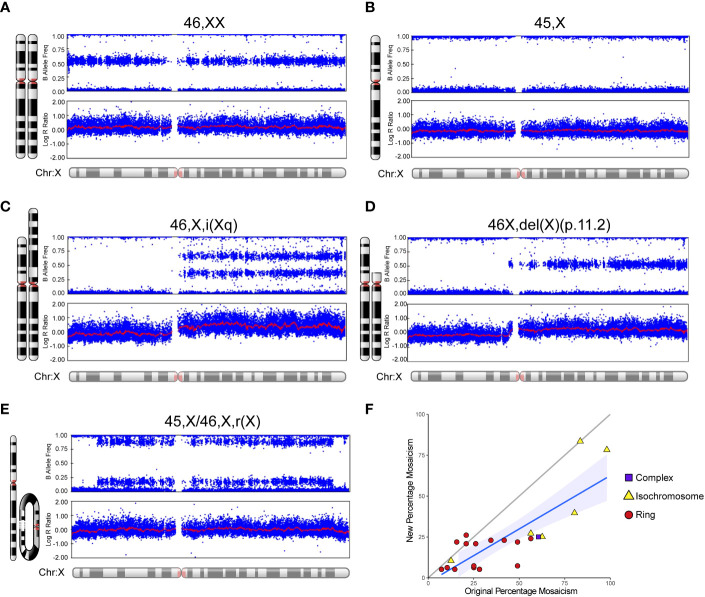
Examples of SNP array data for different karyotypes and mosaic changes over time. Selected karyotype examples are shown for: **(A)** 46,XX; **(B)** 45,X (“monosomy” group); **(C)** 46,X,i(Xq) (“isochromosome” group/isodicentric Xq); **(D)** 46,X,del(X)(p.11.2) (“complex” group); and **(E)** 45,X/46,X,r(X) (“ring” group). **(F)** Scatter plot to show the “new” percentage mosaicism obtained from the SNP array compared to the “original” percentage mosaicism at diagnosis via G-banded karyotype. The percentage mosaicism refers to the percentage of the variant (isochromosome, ring) cell line or chromosome. Array data were analyzed and visualized in Illumina Genome Studio v2.0. Plots were generated of the B-allele frequency (the normalized measure of the allelic intensity ratios of two alleles A, B) and the log R ratio (the normalized measure of signal intensity for each SNP marker, as log2 of the ratio between observed and expected for two copies of the genome). The mean value is represented by a red horizontal line. For B allele frequency, the number of bands seen on the plot minus one usually indicates the number of chromosomes at that given locus. B allele frequencies (BAF) of 0.0, 0.5 and 1.0 are expected in a normal sample, representing AA, AB and BB, respectively. For the log R ratio, a signal clustering around zero shows when the region has two copies; higher or lower signal intensities indicate when there are more or less copies in a genomic region, respectively. Where relevant, the percentage of mosaicism was calculated using the pipeline developed by Conlin et al. (38).

An overview of the study cohort and sub-groups is shown in **Table 1**. Monosomy X (45,X) was present in 75/134 (56.0%) women whereas 44% (59/134) of women had related karyotypes. Typical SNP array outputs for different karyotypes are shown in **Figures 3A–E**, showing haploinsufficiency of PAR1 in all situations. For those women diagnosed with mosaic isodicentric Xq (45,X/46X,i(Xq)) or ring (45,X/46,X,r(X)) karyotypes, the proportion of 45,X cells was often higher on the more recent (“new”) SNP array analysis compared to the original karyotype analysis (Wilcoxon matched-pairs signed rank test P value <0.0001) (**Figure 3F**), although different platforms for assessment were used.

### 3.2 Genetic variability in women with TS

To investigate whether disruption or changes in the X chromosome in TS affect genetic (exome) variation in general (**Figure 1**, *center illustration*), total autosomal and X chromosome variants were compared between cohorts as well as the potential translational impact of any changes.

Out of the 7,558,157 variants identified in the study (**Figure 2**), the mean number of variants *per individual* was approximately 28,000. The standard filtering approach for germline or early somatic events had a combined total of 6,457,085 autosomal variants and 126,335 X chromosome variants. The somatic mosaic filtering approach looked at low variant allele frequency changes between 5-25% and this gave a total of 16,223 autosomal variants and 477 variants on the X chromosome.

#### 3.2.1 Autosomal variants

**Figure 4:**
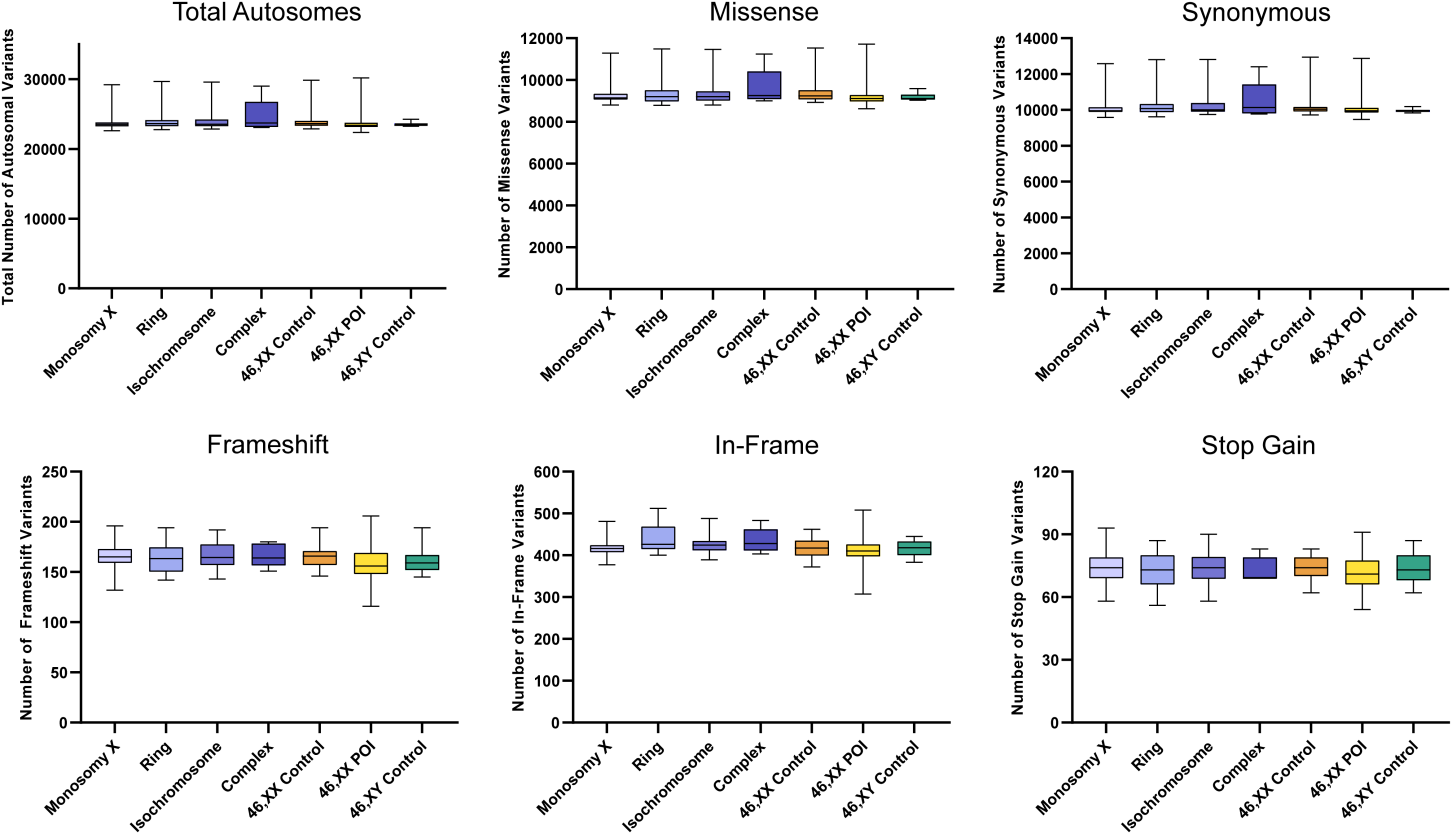
Number of autosomal variants in each cohort and predicted translational impact. The total number of variants are shown in the top left panel. The number of variants predicted to cause missense, synonymous, frameshift, in-frame and stop gain changes are shown subsequently from left to right. Data are represented as box plots showing the lower quartiles, upper quartiles and the median with the whiskers showing the range of the data. No statistically significant differences (p<0.05) were found between any of the cohorts in each analysis (Kruskal-Wallis one-way analysis of variance). Sequencing parameters (read depth, call quality) for all groups are shown in **Supplementary Figure 3A**. POI, primary ovarian insufficiency.

There were no significant differences between the total number of autosomal variants found in the different TS cohorts compared to the different control/comparison cohorts (**Figure 4**). No differences were found in different subcategories of variants. These findings suggest there is no excess genetic variation in autosomes in TS women.

#### 3.2.2 X chromosome variants

**Figure 5:**
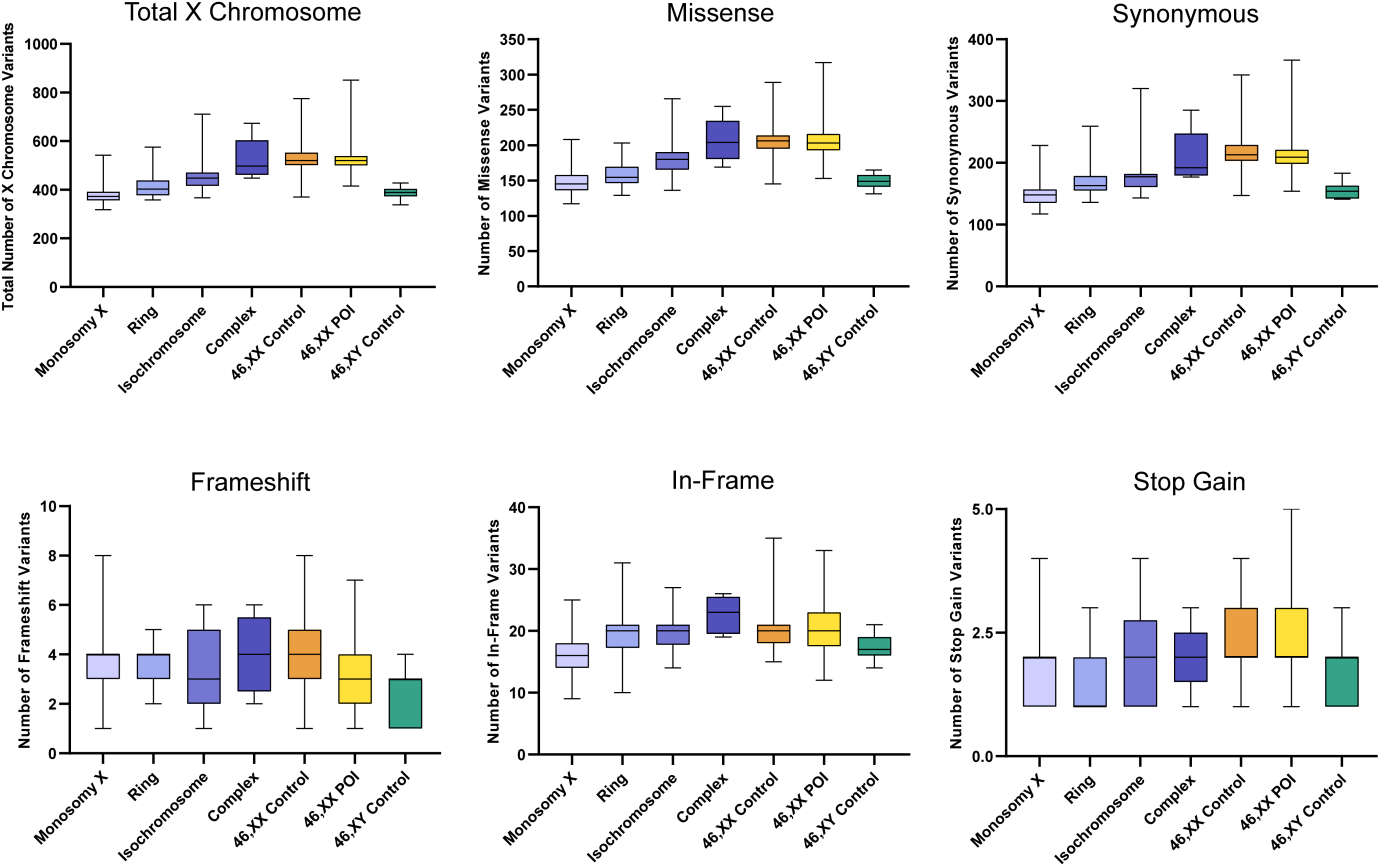
Number of X chromosome variants in each cohort and predicted translational impact. The total number of variants are shown in the top left panel. The number of variants predicted to cause missense, synonymous, frameshift, in-frame and stop-gain changes are shown subsequently from left to right. Data are represented as box plots showing the lower quartiles, upper quartiles and the median with the whiskers showing the range of the data. The number of X chromosome variants appeared proportionate to the amount of X chromosome material. There was no excess of variability in monosomy (45,X) compared to 46,XY controls, or in other TS subcategories compared to 46,XX controls. Statistical differences between groups are shown in **Supplementary Figure 2**. Sequencing parameters (read depth, call quality) for all groups are shown in **Supplementary Figure 3B**. POI, primary ovarian insufficiency.

The total number of X chromosome variants was proportionate to the amount of X chromosome material present in each karyotype (**Figure 5**). For example, the number of X chromosome variants in the monosomy group (45,X) with a single X was very similar to the 46,XY control group, whereas those with isochromosomes had variant numbers approaching 46,XX women or 46,XX POI comparison cohorts. Any significant differences between groups are shown in **Supplementary Figure 2**. Overall, these findings suggest there is no excess genetic variation in X chromosomes in TS women.

#### 3.2.3 Somatic mosaic autosomal and X chromosome variants

**Figure 6:**
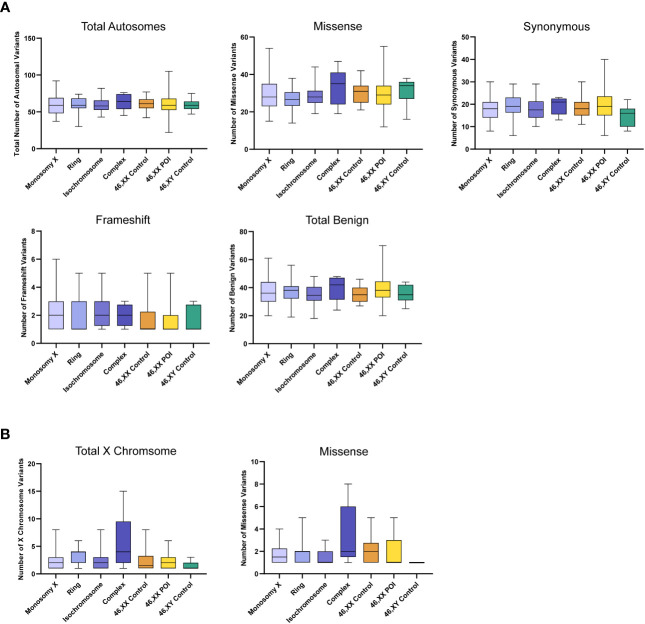
Number of autosomal somatic variants in each cohort and predicted translational impact for somatic variants. **(A)** The total number of somatic autosomal variants are shown in the top left panel. The number of somatic variants predicted to cause missense, synonymous, frameshift, and predicted total benign somatic changes are shows subsequently from left to right. Data are represented as box plots showing the lower quartiles, upper quartiles and the median with the whiskers showing the range of the data. No statistically significance differences (p<0.05) were found between any of the cohorts in each analysis (Kruskal-Wallis one-way analysis of variance) **(B)** The total number of somatic X chromosome variants are shown in the left panel and missense variants are shown on the right. Sequencing parameters (read depth, call quality) for all groups are shown in **Supplementary Figure 4**. POI, primary ovarian insufficiency.

Somatic mosaic changes in the autosomes showed no significant differences between the TS subgroups and the control/comparison cohorts (**Figure 6A**). In general, the number of somatic mosaic variants on the X chromosome was low in all groups studied (**Figure 6B**). Thus, no excess of somatic mosaic variants was observed in leukocyte-derived DNA.

### 3.3 X chromosome genetic variability and phenotype

To address whether common hemizygous variants in X chromosome genes contribute to associated features in TS (**Figure 1**, *right illustration*), we initially focused subsequent analysis only on women with monosomy X (45,X) who had detailed phenotypic data available for DM, obesity, autoimmunity, hypothyroidism, hypertension and cardiac surgery for congenital heart defects. Only women with monosomy X were included in the initial analysis as they have just a single copy of the X chromosome, avoiding any issues with multi-allelic expressed genes and allowing us to focus on hemizygous variants that could act as important drivers of phenotype especially on the background of haploinsufficiency of PAR genes and of genes that escape Xi. As we hypothesized that *common population variants* could have a major effect in this biological context, cohorts of 45,X women with and without a given phenotype (e.g., diabetes versus non-diabetes) were directly compared for enrichment of X chromosome genetic variants that could act as “risk” or “protective” alleles in this situation.

**Figure 7:**
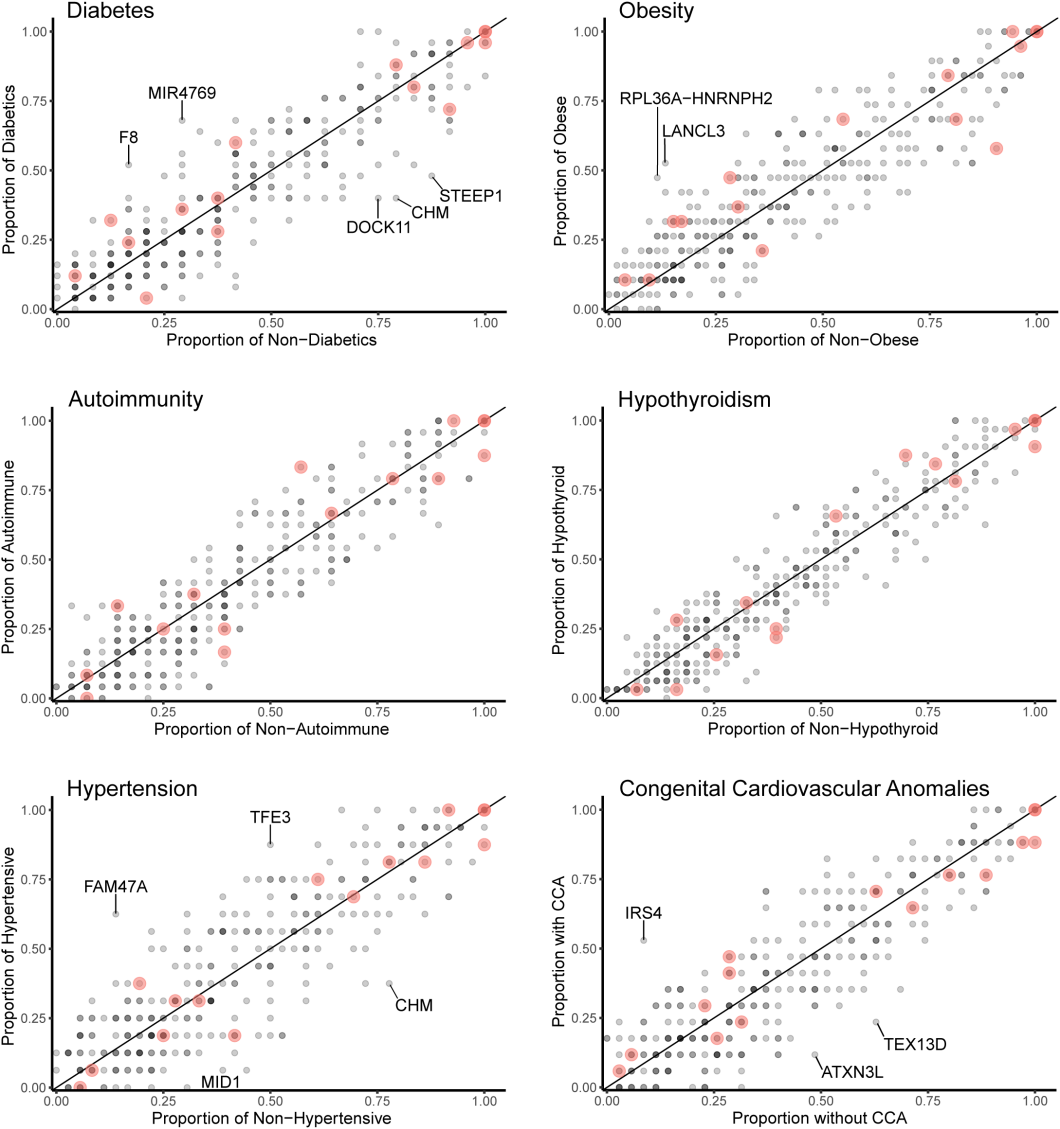
Scatterplots (gene level) of the proportion of X chromosome gene variants in 45,X women with a condition against the proportion of variants in the same gene in those women without the condition. The number of data points at any given coordinate is shown by the intensity of the circle. Red circles indicate genes that are located in the pseudoautosomal regions. Genes that have an effect size greater than 0.35 are labeled. Sequencing parameters (read depth, call quality) for groups are shown in **Supplementary Figure 5**.

**Figure 8:**
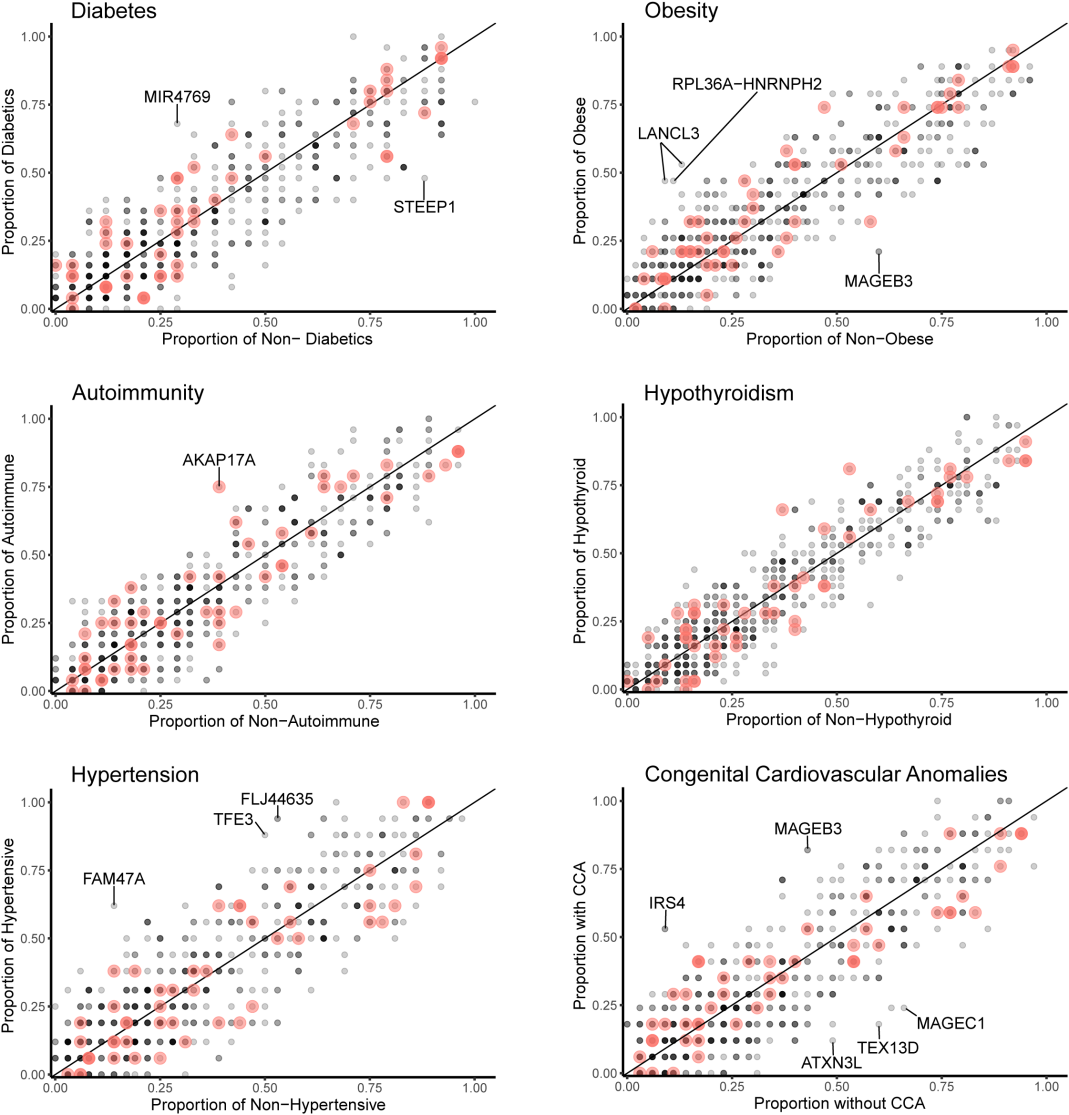
Scatterplots (variant level) of the proportion of X chromosome gene variants in 45,X women with a condition against the proportion of variants in the same gene in those women without the condition. The number of data points at any given coordinate is shown by the intensity of the circle. Red circles indicate genes that are located in the pseudoautosomal regions. Genes that have an effect size greater than 0.35 are labeled. Sequencing parameters (read depth, call quality) for groups are shown in **Supplementary Figure 5**.

Data for gene level analysis of all X chromosome genes containing variants for each major phenotypic condition are shown in **Figure 7**. These are presented as scatterplots of the proportion of gene variants in 45,X women with a condition against the proportion in those without a condition. PAR genes are shown in red. Similar data for variant level analysis are shown in **Figure 8**.

Differences between the proportions of variants in genes in those with and without a condition (“effect size”) are shown in **Table 2** and **Supplementary Table 2**. A positive effect (and odds ratio >1.0) would be expected with a potential “risk” gene (i.e., higher in those with a condition), and a negative effect (odds ratio <1.0) would be expected with a potential “protective” gene (i.e., higher in those without a condition). Data are shown in **Table 2** for genes where effect size was greater than +0.35 or -0.35. Although these genes all showed significant enrichment on individual burden testing (Fisher’s test), none of them were significant when adjusted for multiple testing of X chromosome genes (>600) (Bonferroni correction).

**Table 2:** Proportion of X chromosome genes harboring variants in 45,X women with a condition against the proportion of the same gene harboring variants in those women without the condition.

Gene	Condition	Alt. individuals, with condition	Alt. individuals, without condition	Effect size	OR (95% CI)	Fisher Exact test (p value)	Bonferroni corrected (p.adj)	OMIM	HPA gene expression
*FAM47A*	Hypertension	0.63	0.14	0.49	9.73 (2.2-52)	0.0007	0.23	–	Testis
*IRS4*	Congenital cardiovascular anomaly	0.53	0.09	0.44	11.26 (2.2-80)	0.0008	0.26	Central hypothyroid-ism	Hypothalamus, pituitary, ovary
*LANCL3*	Obesity	0.53	0.13	0.39	0.14 (0.04-0.5)	0.001	0.38	–	Hypothalamus, cerebellum, thalamus
*MIR4769*	Diabetes	0.68	0.29	0.39	4.97 (1.3-21)	0.01	1.00	–	–
*TFE3*	Hypertension	0.88	0.50	0.38	6.76 (1.3-70)	0.01	1.00	XL-LD + pigmentary disorder	Non-specific
*RPL36A-HNRNPH2*	Obesity	0.47	0.11	0.36	0.15 (0.03-0.6)	0.002	0.66	XL-LD (*HNRNPH2*)	Retina, ovary, breast, bone marrow
*F8*	Diabetes	0.52	0.17	0.35	5.22 (1.2-27)	0.02	1.00	Hemophilia A	Heart, tongue, adipose
*DOCK11*	Diabetes	0.40	0.75	-0.35	0.23 (0.05-0.9)	0.02	1.00	–	Adipose, bone marrow, macrophages
*ATXN3L*	Congenital cardiovascular anomaly	0.12	0.49	-0.37	0.15 (0.01-0.8)	0.01	1.00	–	Testis (spermato-genesis)
*MID1*	Hypertension	0.06	0.44	-0.38	0.09 (0.002-0.7)	0.01	1.00	Opitz GBBB syndrome	Cerebellum, colon, heart
*CHM*	Diabetes	0.40	0.79	-0.39	0.18 (0.04-0.7)	0.01	1.00	Choroido-remia	Non-specific
*TEX13D*	Congenital cardiovascular anomaly	0.24	0.63	-0.39	0.19 (0.04-0.8)	0.02	1.00	–	Testis (spermato-genesis)
*STEEP1*	Diabetes	0.48	0.88	-0.40	0.14 (0.02-0.6)	0.01	1.00	XL-LD	Non-specific
*CHM*	Hypertension	0.38	0.78	-0.40	0.18 (0.04-0.7)	0.01	1.00	Choroido-remia	Non-specific

Only women with monosomy X (45,X) and well-defined phenotypic data were included in this analysis. Gene level data are shown. Data are shown where effect size is 0.35 or above. A positive effect size for a condition denotes a potential “risk” allele, whereas a negative effect size denotes a potential “protective” allele. Numbers in the groups analyzed are: diabetes (DM/IGT) n=25, non-diabetes n=24; obese n=19, non-obese=53; hypertension n=16, non-hypertension, n=36; congenital cardiovascular anomaly (CCA) n=17, non-CCA n=35. Bonferroni corrections were made for all genes on the X chromosome where variants were identified (>600 genes). CI, confidence interval; HPA, human protein atlas; OMIM, Online Mendelian inheritance in Man; OR, odds ratio; XL-LD, X-linked learning difficulty.

Despite the lack of significance following multiple corrections (for more than 600 genes), further review of the expression, biological role and clinical associations of these genes was undertaken (**Table 2**). For DM, potential “risk” genes were *MIR4769* and *F8*, whereas “protective” genes were *DOCK11* and *STEEP1*. In the obesity group, *LANCL3*, a hypothalamic expressed gene associated with carbohydrate metabolism (42) and *RPL36A−HNRNPH2* were potential risk genes. *FAM47A* and *TFE3* were potential risk genes for hypertension, whereas *IRS4*, an insulin signaling-pathway gene associated with central hypothyroidism (43), potentially linked with cardiac defects. *CHM* was potentially protective for DM and hypertension. Most of these gene level changes were the result of just one or two single nucleotide variants in a gene, most of which were individually not likely to have major functional effects based on function prediction algorithms (SIFT, PolyPhen), splice analysis or Combined Annotation Dependent Depletion (CADD) scores (**Supplementary Table 2**).

No PAR genes were enriched in the analysis although p.Pro500Ala (X-1601004:C-G) in *AKAP17A*, linked to autoimmunity and showed non-adjusted significance in the variant level analysis (variant allele frequency 0.75 in autoimmune and 0.39 in non-autoimmune; effect size 0.36; gnomAD males 0.54; p-value <0.01, adj.p-value 1.0) (**Figure 8**; **Supplementary Table 2**). Extending the analysis of just PAR genes to a larger group of women with other karyotypes (**Supplementary Figure 6**) did not strengthen association of this variant with autoimmunity (AI n=42, non-AI n=53) (variant allele frequency 27/42 = 0.64 in AI and 31/53 = 0.58 in non-AI, effect size 0.06, p-value 0.18, adj.p-value 1). Analysis of PAR gene variants in relation to phenotype for a larger group of women with different TS-associated karyotypes is shown in **Supplementary Materials** (**Supplementary Figures 6**–**8**; **Supplementary Tables 3**, **4**). Hearing loss data are shown in **Supplementary Tables 5**, **6** and **Supplementary Figure 9**.

### 3.4 *TIMP3* variants and congenital cardiovascular anomalies

Focused analysis of variants in *TIMP3* in relation to cardiac anomalies (CCA n=22, non-CCA n=73) revealed a significant enrichment of the 22:32857305:C-T (rs11547635) variant in the CCA group (MAF CCA 0.14 versus MAF non-CCA 0.03, p-value <0.02) (**Table 3**). This variant is predicted to be synonymous (p.S83S) with a MAF of 0.09 in gnomAD, but has been reported previously in association with CCA in TS in two datasets (**Table 3**). No effect on splicing was predicted using several algorithms (https://spliceailookup.broadinstitute.org/). A proposed combined effect of this *TIMP3* variant with *TIMP1* copy number (1.0) for cardiac risk (35) could not be explored further as we excluded women with 46,XX lines (and higher *TIMP1* copy number) from the cohort. Notably, there was a statistically higher proportion of women with monosomy X in the CCA group, and lower proportion of those with ring chromosomes (see **Supplementary Figure 6** and legend).

**Table 3:** Common variants in *TIMP3* in relation to congenital cardiac anomalies (CCA).

*TIMP3* Variant	Protein	gnomAD MAF	CCA MAF (alleles) (n=22)	Non-CCA MAF (alleles) (n=73)	Fishers Exact test (p-value)	Previously reported CCA MAF	Previously reported Non-CCA MAF
22:32857293:T-C	p.H83H	0.61	0.57 (25/44)	0.51 (75/146)	0.61	0.50 (Corbitt et al., 2018) (35) 0.44 (Corbitt et al., 2019) (36)	0.50 (Corbitt et al., 2018) (35) 0.51 (Corbitt et al., 2019) (36)
22:32857305:C-T*	p.S87S	0.09	0.14 (6/44)	0.03 (5/146)	0.02	0.14 (Corbitt et al., 2018) (35) 0.12 (Corbitt et al., 2019) (36)	0.06 (Corbitt et al., 2018) (35) 0.04 (Corbitt et al., 2019) (36)

gnomAD version (v3.1), GRCh38; *also known as rs11547635. Congenital cardiac anomaly (CCA) group included 22 individuals and non-CCA group included 73 individuals. MAF, minor allele frequency.

Recently, a variant in the candidate cardiac risk gene *CRELD1* (c.9943412, G>A) has been reported to be associated with CCA in women with TS (44). We could not replicate this finding in our cohort (p-value 0.13), potentially due to our smaller sample size, and low allele frequency of this variant.

## 4 Discussion

TS is an important condition with many long-term associated features but the underlying mechanism (or mechanisms) leading to these comorbidities is still not clear. Often this is assumed to be haploinsufficiency of PAR genes on the X chromosome, but other subtle mechanisms related to X gene dosage or autosomal/X-chromosome “ripple effects” may be important (2). By using large-scale high-throughput exome sequencing of a cohort of 134 adult Turner women with a range of long-term associated conditions, we have been able to start to address some of these questions.

Before engaging in in-depth genomic analysis, our first goal was to assess the karyotypes of this cohort of women using a SNP array approach, rather than the traditional G-banded karyotyping and 30 cell mosaic screen. Using this platform, a current karyotype was determined for all participants. Three women (3/134, 2.2%) originally reported to have non-mosaic isochromosomes (46,X, i(Xq)) were found to have a low-level mosaic 45,X line present on array (45,X/46X,i(Xq)). Three additional women had other changes in karyotype, but original records were limited, or small numbers of cells screened. Overall, SNP array proved to be a useful approach to assessing karyotype for this cohort and there was concordance with original recorded karyotype in more than 95% individuals.

In the SNP array dataset, mosaicism levels for ring and isochromosomes were calculated using the approach reported by Conlin et al. (38). As expected, ring chromosomes were present in a low percentage as they are only viable together with a 45,X mosaic line, whereas isochromosome proportions were more variable and generally higher. When the “original” mosaic percentage of these ring and isochromosome cell lines reported by G-banded karyotype/mosaic screen was compared to the “new” array-derived mosaicism percentage in the same individual, a lower proportion of ring and isochromosome cell lines was seen, and higher proportion of 45,X line. This observation may represent a clonal selection advantage for the 45,X line with time especially in the hematopoietic system, and dynamic changes over time have been reported in forms of revertant mosaicism (45) and even related to loss of the Y chromosome with age (46). Alternatively, these findings may reflect the different methodologies used; historic mosaic screens were undertaken on relatively small numbers of cells, whereas SNP array derives a mosaicism level based on signal intensity. Unfortunately, we were not able to repeat traditional karyotype mosaic screens on this cohort.

Having defined karyotypes in the cohorts, targeted exome sequencing was used to test the hypothesis of whether changes in the X chromosome with TS are associated with differences in global genomic variability either across the autosomes or in the X chromosome itself. It has been hypothesized that haploinsufficiency of key X chromosome genes could have “ripple effects” affecting transcription, translation, splicing or methylation/chromatin across the genome (2, 21–23, 27, 29–34, 47, 48). We extended this concept further to investigate whether there are global differences in genetic variability in TS, potentially as a consequence of loss of a DNA proofreading or repair gene on the X chromosome (**Figure 1**). With the reduction in cost and ease of throughput for exome sequencing, we looked at exome variability in the entire TS cohort and subgroups, and compared the data to 46,XX controls, 46,XY controls and additional comparison cohort of 46,XX women with POI. Using this approach, no significant differences in autosomal genomic variability were found between these groups and X chromosome genomic variability tracked very clearly with the amount of X chromosome material present. A filtering approach to detect lower-level potential somatic mosaic events was developed, as somatic variability in DNA derived from rapidly replicating blood cell lineages is an ideal system to assess this. However, no significant changes between the groups were seen. These data show that global genetic (exome) variability in TS is likely to be unaffected.

In the past 35 years, investigations of associations between genetic variability and phenotype in TS initially focused on parent of origin effects of the X chromosome, based on the potential existence of imprinted X chromosome genes (**Supplementary Table 7**). Other studies considered the influence of mostly non-coding single nucleotide variants on either specific features of TS, or more general features (e.g., bone mineral density (*VDR*, *ESR1*); response to growth hormone treatment (*GHRd3*)) (**Supplementary Table 7**). Data for coding sequence changes, especially for X chromosome genes, are very limited.

Here, we hypothesized that, whilst haploinsufficiency of key X chromosome genes is often thought to be an important underlying pathogenic event in TS, monosomy X is a unique biological situation where common genetic variability in X chromosome genes could be “uncovered” by the unexpected loss of the second sex chromosome, and these variants could act as strong “drivers” of phenotype. Women with TS can have a broad range of conditions such as diabetes mellitus, obesity, autoimmune disease and hypothyroidism and hypertension, as well as developmental features such as congenital heart defects. Research efforts to try to understand the biological basis of some of these conditions are very important in order to develop new and personalized treatments for the TS community. We therefore investigated whether any gene level or variant level changes in any gene on the X chromosome were significantly enriched in monosomy X women with a specific condition compared to those without it (“risk” allele), or conversely whether changes could be found more often in women without a given condition compared to those with it (“protective” allele).

Using this approach, several potentially enriched genes and variants were found, but the effect size was limited (all less than 0.5 between groups) and statistical differences did not withstand multiple comparison testing. Furthermore, biological correlations of proposed gene function with phenotype were not obvious, except in the case of obesity and *LANCL3*. This gene has been linked to carbohydrate metabolism/high fat diet induced obesity in rats, is expressed in the hypothalamus and has been shown to escape X inactivation in TS, so further investigation may be warranted (32, 42). The only PAR gene that emerged was a variant in *AKAP17A* (X:1601004:C-G) associated with autoimmunity. This gene has been implicated in inflammatory bowel disease (49) but this variant was predicted to be benign.

Given a previously reported association between the 22:32857305:C-T (rs11547635) variant in *TIMP3* and congenital cardiovascular anomalies in two cohorts of women with TS, we undertook a specific analysis of *TIMP3* and CCA in our study group. *TIMP3* encodes Tissue Inhibitor of Metalloprotease 3, an extracellular matrix protein involved in angiogenesis and cardiac remodeling (50, 51). The *TIMP3* gene is located on chromosome 22. It is hypothesized that loss of *TIMP1* (on the X chromosome) coupled with variations in *TIMP3* can predispose to CCA in women with TS, in an autosomal “two-hit” hypothesis (2, 35, 36). Very recently, the rs11547635 variant (22:32857305:C-T) in TIMP3 has also been associated with aortic regurgitation in a longer term follow up study of Turner women (52). Notably, we were able to replicate the enrichment of this 22:32857305:C-T variant with CCA in our cohort (**Table 3**). Indeed, MAF data were very similar to previous reports (35, 36). This finding is important not only for independently reproducing other studies, but it also demonstrates that rare variants may influence phenotype in a two-hit manner, which is the basis of our X chromosome gene hypothesis. Furthermore, the MAF for this *TIMP3* variant is relatively low (CCA 0.14 versus non-CCA 0.03) and effect size small (0.11). Our X chromosome variant analysis was far more stringent, focusing on an effect size cut off at +/- 0.35 and adjusting for multiple comparisons for all X genes. Given the *TIMP3* data, several of the variants we detected in the X chromosome may be biologically significant. This could be addressed in the future in larger cohorts, combining our datasets with additional cohorts.

This study has several limitations. Firstly, clinical associated phenotypes can change with time, so an individual currently classed as non-diabetic may develop diabetes later in life. Indeed, some phenotypes may be influenced by other factors such as family history, have multifactorial origins (such as hypertension, obesity or combined autoimmune (Type 1) and metabolic effects (Type 2) in diabetes mellitus), or be inter-dependent (e.g., DM/BMI; hypothyroidism/autoimmunity). In order to improve phenotype accuracy, gold standard approaches and detailed re-evaluations were undertaken, such as oral glucose tolerance testing to capture women with undiagnosed diabetes as well as those on established treatment. Only women with robust phenotypic data were included for analysis. Secondly, variant numbers can be influenced by sequencing quality (quality, depth) and batch effects. We compensated for this by processing all samples at the same time and using robotic approaches to undertake library preparations. Third, the numbers of individuals in the phenotype groups for X chromosome variant analysis were relatively small and the power of burden testing when adjusted for multiple comparisons would have been stronger if more individuals had been included. Our hypothesis was that *common* population variants in X chromosome genes are exposed and contribute to phenotype. However, the power to detect statistical differences is dependent on sample number, and potentially important changes with a smaller effect size may have been missed. As discussed above, the potential influence of *TIMP3* on congenital cardiac phenotypes was replicated, even for a variant of low allele frequency and small effect size. Fourth, some studies have suggested that tissue mosaicism for a covert 46,XX or 46,XY cell line may influence phenotype (2). In our study, any individuals with evidence of a 46,XX or 46,XY line in the blood were excluded, and two independent samples (karyotype, SNP array) were analyzed usually many years apart, so – whilst tissue specific mosaicism cannot be excluded – we feel it is unlikely. Finally, we did not undertake analysis of DNA methylation effects across the genome, or transcriptional/translational networks. This would require specific methylation assays or RNA studies and would still be limited to analysis of leukocyte profiles unless other tissues of interest were available.

Despite these limitations, this is a major hypothesis-based study using SNP array and exome sequencing in one of the largest populations of women with TS studied at a detailed genetic level to date. This work highlights how rapidly advancing technologies can increasingly be applied at scale to address key questions in well-characterized clinical cohorts, with the aim of developing more specific or personalized approaches to treatment for associated conditions in the future.

## Data availability statement

The datasets presented in this study can be found in online repositories. The names of the repository/repositories and accession number(s) can be found below: https://doi.org/10.17605/OSF.IO/AJBEP, Open Science Framework (53).

## Ethics statement

The studies involving humans were approved by NRES Committee London-Chelsea (16/LO/0682). The studies were conducted in accordance with the local legislation and institutional requirements. The participants provided their written informed consent to participate in this study.

## Author contributions

JS, GC and JA conceptualized the study. JS, AP-C, SM-B, MI, ID, FB, TB, GK, GC and JA undertook data curation. MI and JS undertook formal data analysis. GC and JA were involved in funding acquisition. AP-C, GC and JA undertook investigation, diagnosis, and management. GC and JA oversaw project administration and supervision. MI and ID undertook validation. JS and FB were responsible for data visualization. JS, FB and JA wrote the original draft with input from GC. All authors were involved in reviewing and editing the final manuscript. GC and JA had full access to all data in the study and had final responsibility for the decision to submit for publication. All authors contributed to the article and approved the submitted version.
