# Supplementary material for: Analysis of genetic variability in Turner syndrome linked to long-term clinical features
Source: Front Endocrinol (Lausanne). 2023 Sep 20;14:1227164. doi: 10.3389/fendo.2023.1227164 (PMC10548239; doi:10.3389/fendo.2023.1227164)
Supplement: Supplementary file 1 [file DataSheet_1.pdf]

Supplementary Material

Analysis of genetic variability in Turner Syndrome linked to long-term clinical features

Jenifer P. Suntharalingham, Miho Ishida, Antoinette Cameron-Pimblett, Sinead M. McGlacken-Byrne, Federica Buonocore, Ignacio Del Valle, Gaganjit Madhan Kaur, Tony Brooks, Gerard S. Conway†, John C. Achermann†*

*** Correspondence:** J. C. Achermann: j.achermann@ucl.ac.uk

† denotes equal contribution

Supplementary Table 1. Overview of post-hoc power calculations for effect size 0.35.

Group	n with condition	Alt allele with condition	n without condition	Alt allele without condition	Effect size	alpha	beta	Power
Diabetes mellitus	25	0.48	24	0.13	0.35	0.05	0.23	77%
Obesity	19	0.48	53	0.13	0.35	0.05	0.14	86%
Autoimmunity	24	0.48	28	0.13	0.35	0.05	0.28	80%
Hypothyroidism	32	0.48	43	0.13	0.35	0.05	0.08	92%
Hypertension	16	0.48	36	0.13	0.35	0.05	0.23	77%
CCA	17	0.48	35	0.13	0.35	0.05	0.22	78%
Hearing loss	14	0.48	33	0.13	0.35	0.05	0.28	72%

Post-hoc power calculations are based on the final numbers in each group, for the monosomy only analysis of X-chromosome gene variants. The allele range for 0.35 effect size is given around a population minor allele frequency of 0.3 (i.e., 0.48, 0.13). To reach a power of 80% the following effect sizes would be required: diabetes mellitus, 0.36; hypertension, 0.36; CCA, 0.36; hearing loss, 0.38. CCA, congenital cardiac anomaly.

Supplementary Table 2. Proportion of X chromosome gene variants in 45,X women with a condition against the proportion of variants in the same gene in those women without the condition.

Gene	Position and Change	Condition	Alt. allele with condition	Alt. allele without condition	gnomAD (v3.1.2) VAF (46,XY)	Effect Size	Odds ratio (95% CI)	Fisher's Exact test (p value)	Bonferroni corrected (p.adj)	HGVSp	Variant effect	PolyPhen	SIFT	CADD	*Other in silico* predic-tions (REVEL; SpliceAI)
FAM47A	X:34131150 C-A	Hypertension	0.62	0.14	0.22	0.48	9.73 (2.2-52)	0	0.49	p.Ala377Ser	Missense	Benign	Tole-rated	0.28	REVEL: 0.037; SpliceAI: 0.00
IRS4	X:108733710 G-C	Congenital Cardiovascular Anomaly	0.53	0.09	0.36	0.44	11.3 (2.2-80)	0	0.56	p.His879Asp	Missense	Benign	Tole-rated	0.72	REVEL: 0.068; SpliceAI: 0.00
IRS4	X:108736282 C-T	Congenital Cardiovascular Anomaly	0.53	0.09	0.36	0.44	11.3 (2.1-80)	0	0.56	p.Ala21Ala	Synonymous	NA	NA	8.40	SpliceAI: 0.00
FLJ44635	X:72159852 T-C	Hypertension	0.94	0.53	0.56	0.41	12.9 (1.6-596)	0	1	Non-coding[A]	Non-coding gene	NA	NA	7.36	NA
FLJ44635	X:72160003 C-T	Hypertension	0.94	0.53	0.56	0.41	12.9 (1.6-596)	0	1	Non-coding[B]	Non-coding gene	NA	NA	3.68	NA
LANCL3	X:37659553 C-T	Obesity	0.53	0.13	0.28	0.40	0.14 (0.04-0.5)	0	0.8	p.Ser263Ser	Synonymous	NA	NA	5.70	SpliceAI: 0.00
MAGEB3	X:30236244 G-A	Congenital Cardiovascular Anomaly	0.82	0.43	0.42	0.39	6.0 (1.3-38)	0.01	1	p.Arg107His	Missense	Benign	Tole-rated	0.001	REVEL: 0.062; SpliceAI: 0.00
MAGEB3	X:30236413 A-G	Congenital Cardiovascular Anomaly	0.82	0.43	0.41	0.39	6.0 (1.3-38.4)	0.01	1	p.Val163Val	Synonymous	NA	NA	1.55	SpliceAI: 0.00

Supplementary Table 2 - continued

Gene	Position and Change	Condition	Alt. allele with condition	Alt. allele without condition	gnomAD (v3.1.2) VAF (46,XY)	Effect Size	Odds ratio (95% CI)	Fisher's Exact test (p value)	Bonferroni corrected (p.adj)	HGVSp	Variant effect	PolyPhen	SIFT	CADD	*Other in silico* predic-tions (REVEL; SpliceAI)
MIR4769	X:47585586 T-C	Diabetes	0.68	0.29	0.46	0.39	5.0 (1.3-21)	0.01	1	Complex[C]	Synonymous	NA	NA	6.71	REVEL: 0.018; SpliceAI: 0.01 (acceptor gain)[D]
LANCL3	X:37668406 T-C	Obesity	0.47	0.09	0.15	0.38	0.12 (0.03-0.5)	0	0.67	p.Leu381Pro	Missense	Benign	NA	4.79	REVEL: 0.021; SpliceAI: 0.06 (donor gain)[D]
TFE3	X:49030563 T-C	Hypertension	0.88	0.5	0.35	0.38	6.8 (1.3-70)	0.01	1	p.Val441Val	Synonymous	NA	NA	1.52	SpliceAI: 0.00
AKAP17A	X:1601004: C-G	Autoimmunity	0.75	0.39	0.54	0.36	4.5 (1.2-19)	0.01	1	p.Pro500Ala	Missense	Benign	Tole-rated (LC)	0.24	REVEL: 0.038; SpliceAI: 0.00
RPL36A-HNRNPH 2	X:101390479 T-C	Obesity	0.47	0.11	0.32	0.36	0.15 (0.03-0.6)	0	1	p.Glu24Gly (BTK)	Missense	Benign	NA	11.8	NA
ATXN3L	X:13318940 C-T	Congenital Cardiovascular Anomaly	0.12	0.49	0.51	-0.37	0.15 (0.01-0.8)	0.01	1	p.Gly332Asp	Missense	Benign	Tole-rated (LC)	0.21	REVEL: 0.193; SpliceAI: 0.01 (donor gain)
MAGEB3	X:30236244 G-A	Obesity	0.21	0.6	0.42	-0.39	5.6 (1.5-26)	0.01	1	p.Arg107His	Missense	Benign	Tole-rated	0.001	REVEL: 0.062; SpliceAI: 0.00

Supplementary Table 2 - continued

Gene	Position and Change	Condition	Alt. allele with condition	Alt. allele without condition	gnomAD (v3.1.2) VAF (46,XY)	Effect Size	Odds ratio (95% CI)	Fisher's Exact test (p value)	Bonferroni corrected (p.adj)	HGVSp	Variant effect	PolyPhen	SIFT	CADD	*Other in silico* predic-tions (REVEL; SpliceAI)
MAGEB3	X:30236413 A-G	Obesity	0.21	0.6	0.41	-0.39	5.6 (1.5-26)	0.01	1	p.Val163Val	Synonymous	NA	NA	1.55	SpliceAI: 0.00
MAGEC1	X:141906127 C-G	Congenital Cardiovascular Anomaly	0.24	0.63	0.46	-0.39	0.19 (0.04-0.8)	0.02	1	p.Ser241Ser	Synonymous	NA	NA	2.02	SpliceAI: 0.00
STEEP1 (*CXorf56)*	X:119544401 G-A	Diabetes	0.48	0.88	0.59	-0.40	0.14 (0.02-0.6)	0.01	1	p.Gly125Gly	Synonymous	NA	NA	5.01	SpliceAI: 0.00
MAGEC1	X:141906119 C-T	Congenital Cardiovascular Anomaly	0.24	0.66	0.25	-0.42	0.17 (0.03-0.7)	0.01	1	p.Pro239Ser	Missense	Benign	Tole-rated (LC)	0.04	REVEL: 0.02; SpliceAI: 0.00
TEX13D	X:124333765 T-C	Congenital Cardiovascular Anomaly	0.18	0.6	0.43	-0.42	0.15 (0.02-0.7)	0.01	1	p.Leu283Pro	Missense	Benign	Tole-rated	0.02	SpliceAI: 0.00

Only women with monosomy X (45,X) and well-defined phenotypic data were included in this analysis. Variant level data are shown - where number of alleles are counted. A positive effect size for a condition denotes a potential "risk" allele, whereas a negative effect size denotes a potential "protective" allele. Numbers in the groups analyzed are: diabetes (DM/IGT) n=25, non-diabetes n=24; obesity n=19, non-obesity 53; autoimmunity n=24, non-autoimmunity n=28; hypertension n=16, non-hypertension, n=36; congenital cardiovascular anomaly (CAA) n=17, non-CAA n=35. Bonferroni corrections were made for all genes on the X chromosome where variants were identified. Alt., alternative (minor); CADD, Combined Annotation Dependent Depletion; CI, confidence interval; HGVSp, Human Genome Variation Society protein; LC, low confidence; NA, not applicable; REVEL, Rare Exome Variant Ensemble Learner; SIFT, Sorting Intolerant From Tolerant; VAF, Variant Allele Frequency.

[A]Falls in two transcripts on two genes: BX119917.1 ENST00000432517.1 Ensembl canonical transcript for BX119917.1, HGVScn.8T>C **AND** NHSL2, ENST00000623354.1, HGVScn.371T>C

[B]Non coding transcript exon AND NHSL2, ENST00000623354.1, HGVScn.522C>T, BX119917.1, ENST00000432517.1, Ensembl standard transcript for BX119917.1, HGVScn.159C>T

[C]This variant falls on 7 transcripts in 3 genes (TIMP1 =p.Phe124Phe; SYN1=c.775-8085A>G)

[D]variant has multiple SpliceAI scores

Supplementary Table 3. Proportion of PAR genes harboring variants in women with TS with a condition against the proportion of PAR genes harboring variants in those women without the condition.

Gene	Condition	Alt. individuals, with condition*	Alt. individuals, without condition*	Effect size	OR (95% CI)	Fisher Exact test (p value)	Bonferroni corrected (p.adj)	OMIM	HPA gene expression
CRLF2	Autoimmunity	0.93	0.74	0.19	4.60 (1.2-26.9)	0.017	0.3	–	Tissue enhanced in bone marrow, lymphoid tissue
CRLF2	Hypothyroidism	0.93	0.79	0.14	3.35 (1.0-14.5)	0.032	0.58	–	Tissue enhanced in bone marrow, lymphoid tissue
ASMT	Congenital cardiovascular anomaly	0.18	0.04	0.14	5.07 (0.8-37.7)	0.048	0.86	–	Low tissue specificity
ASMTL	Congenital cardiovascular anomaly	0.86	0.99	-0.12	0.09 (0.002-1.2)	0.038	0.68	–	Non- specific
CSF2RA	Obesity	0.74	0.93	-0.18	4.34 (1.3-15.2)	0.012	0.22	Surfactant metabolism dysfunction, pulmonary, 4	Placenta

Only women with Turner syndrome and well-defined phenotypic data were included in this analysis. *Gene level data are shown - where unique individuals having a variant in a gene are counted and not the number of alleles. A positive effect size for a condition denotes a potential “risk” allele, whereas a negative effect size denotes a potential “protective” allele. Numbers in the groups analyzed are obesity n=35, non-obesity=96; autoimmunity n=42, non-autoimmunity n=53; hypothyroidism n=54, non-hypothyroidism, n=80; congenital cardiovascular anomaly (CAA) n=22, non-CAA n=73. Bonferroni corrections were made for all genes on the X chromosome where variants were identified. HPA, human protein atlas; OMIM, Online Mendelian inheritance in Man; OR, odds ratio; TS, Turner Syndrome.

Supplementary Table 4. Proportion of PAR gene variants in women with Turner syndrome with a condition against the proportion of variants in the same gene in those women without the condition.

Gene	Position and Change	Condition	Alt. allele with condition	Alt. allele without condition	gnomAD (v3.1.2) VAF (46,XY)	Effect Size	Odds ratio (95% CI)	Fisher's Exact test (p value)	Bonferroni corrected (p.adj)	HGVSp	Variant effect	Poly-Phen	SIFT	CADD	Other in silico predic-tions (REVEL; SpliceAI)
CRLF2	X:1198742: C-T	Autoimmunity	0.43	0.21	0.4467 potential low qual site	0.22	3.65 (1.3-10.4)	0.01	0.91	NA	NA	NA	NA	NA	NA
CD99	X:2726261: C-T	Hypertension	0.33	0.12	0.1117	0.21	3.69 (1.1-12.8)	0.02	1	p.Ala121Ala	Synonymous	NA	NA	0.893	SpliceAI: 0.00
CD99	X:2726261: C-T	Hypothyroidism	0.28	0.1	0.1117	0.18	3.43 (1.2-10.2)	0.01	1	p.Ala121Ala	Synonymous	NA	NA	0.893	SpliceAI: 0.00
SLC25A6	X:1389431: G-A	Autoimmunity	0.33	0.17	0.3071	0.16	3.0 (1.0-9.2)	0.03	1	p.Phe136Phe	Synonymous	NA	NA	9.34	SpliceAI: 0.0100
CD99	X:2726261: C-T	Autoimmunity	0.26	0.11	0.1117	0.15	3.34 (1.0-12.3)	0.03	1	p.Ala121Ala	Synonymous	NA	NA	0.893	SpliceAI: 0.00
SLC25A6	X:1389690: T-C	Hypothyroidism	0.24	0.1	0.121	0.14	2.83 (0.991-8.6)	0.05	1	p.Gln50Arg	Missense	Benign	Tole-rated	15.9	REVEL: 0.260 SpliceAI: 0.00
PPP2R3B	X:361426: A-G	Autoimmunity	0.69	0.58	0.6181	0.11	2.66 (1.0-8.0)	0.04	1	p.Asp163Asp	Synonymous	NA	NA	0.923	SpliceAI: 0.00
AKAP17A	X:1593471: G-A	Congenital Cardiovascular Anomaly	0.09	0	0.02145	0.09	Inf (0.6-Inf)	0.05	1	p.Ala3Ala	Synonymous	NA	NA	9.81	SpliceAI: 0.00
CSF2RA	X:1309528: G-T	Hypothyroidism	0.02	0.12	0.1466	-0.1	0.13 (0.003-1.0)	0.05	1	p.Arg358Leu	Missense	Benign	NA	0.989	REVEL: 0.0420; SpliceAI: 0.00

Supplementary Table 4 - continued

Gene	Position and Change	Condition	Alt. allele with condition	Alt. allele without condition	gnomAD (v3.1.2) VAF (46,XY)	Effect Size	Odds ratio (95% CI)	Fisher's Exact test (p value)	Bonferroni corrected (p.adj)	HGVSp	Variant effect	Poly-Phen	SIFT	CADD	Other in silico predic-tions (REVEL; SpliceAI)
IL3RA	X:1348511: A-C	Obesity	0	0.11	0.09033	-0.11	Inf (0.97-Inf)	0.04	1	p.Pro88Pro	Synonymous	NA	NA	1.57	SpliceAI: 0.210
CRLF2	X:1198738: NA-ACAT	Obesity	0.11	0.31	–	-0.2	3.49 (1.1-14.8)	0.02	1	NA	NA	NA	NA	NA	NA
PLCXD1	X:284193: T-C	Congenital Cardiovascular Anomaly	0.64	0.85	0.7828	-0.21	0.32 (0.09-1.1)	0.04	1	p.Gly2Gly	Synonymous	NA	NA	2.68	SpliceAI: 0.0100
IL3RA	X:1381116: T-C	Hypertension	0.07	0.29	0.1701	-0.22	0.19 (0.02-0.9)	0.03	1	NA	NA	NA	NA	1.3	SpliceAI: 0.00
CSF2RA	X:1288904: AT-NA	Obesity	0.34	0.57	–	-0.23	2.55 (1.1-6.3)	0.03	1	NA	NA	NA	NA	NA	NA
ASMTL	X:1427983: A-G	Congenital Cardiovascular Anomaly	0.55	0.78	0.6614	-0.23	0.34 (0.1-1.1)	0.05	1	p.Arg216Arg	Synonymous	NA	NA	2.15	SpliceAI: 0.00
ASMTL	X:1427899: G-A	Congenital Cardiovascular Anomaly	0.55	0.79	0.6492	-0.24	0.31 (0.1-1.0)	0.03	1	p.Gly244Gly	Synonymous	NA	NA	1.95	SpliceAI: 0.0100
CRLF2	X:1198742: C-T	Congenital Cardiovascular Anomaly	0.05	0.38	0.4467 potential low qual site	-0.33	0.08 (0.002-0.5)	0	0.42	NA	NA	NA	NA	1.3	SpliceAI: 0.00

Only women with Turner syndrome and well-defined phenotypic data were included in this analysis. Variant level data are shown – where number of alleles are counted. All variants with an unadjusted P value<0.05 are included. A positive effect size for a condition denotes a potential "risk" allele, whereas a negative effect size denotes a potential "protective" allele. Numbers in the groups analyzed are obesity n=35, non-obesity=96; autoimmunity n=42, non-autoimmunity n=53; hypothyroidism n=54, non-hypothyroidism, n=80; congenital

Supplementary Table 4 - continued

cardiovascular anomaly (CAA) n=22, non-CAA n=73. Bonferroni corrections were made for all genes on the X chromosome where variants were identified. Alt., alternative (minor); CADD, Combined Annotation Dependent Depletion; CI, confidence interval; HGVSp, Human Genome Variation Society protein; LC, low confidence; NA, not applicable; REVEL, Rare Exome Variant Ensemble Learner; SIFT, Sorting Intolerant From Tolerant; VAF, Variant Allele Frequency.

Supplementary Table 5. Proportion of X chromosome genes in 45,X women with hearing loss* against the proportion of variants in the same gene in those women without hearing loss.

Gene	Condition	Alt. individuals with condition	Alt. individuals without condition	Effect size	OR (95% CI)	Fisher Exact test (p value)	Bonferroni corrected (p.adj)	OMIM	HPA gene expression
ARSD	Hearing Loss	0.86	0.42	0.43	7.79 (1.4 - 82.9)	0.01	1		Liver, kidney, pancreas
SLC16A2	Hearing Loss	0.86	0.42	0.43	7.79 (1.4 - 82.9)	0.01	1	Allan-Herndon-Dudley syndrome (*MCT8* deficiency)	Liver, adrenal gland
GAGE12J	Hearing Loss	0.79	0.36	0.42	6.15 (1.3 - 41.2)	0.01	1		Testis (spermato-genesis)
TLR8	Hearing Loss	0.71	0.30	0.41	5.51 (1.2 - 30.3)	0.01	1	Immuno-deficiency 98 with auto-inflammation, X-linked	Spleen, lymph node, lung
CFP	Hearing Loss	0.50	0.12	0.38	6.87 (1.3 - 42.2)	0.01	1	Properdin deficiency, X-linked	Bone marrow, spleen
IL1RAPL1	Hearing Loss	0.86	0.48	0.37	6.14 (1.1 - 65.1)	0.02	1	Intellectual developmental disorder, X-linked 21	Brain
SNX12	Hearing Loss	0.86	0.52	0.34	5.46 (1.0 - 57.7)	0.05	1		Non-specific
BRWD3	Hearing Loss	0.00	0.27	-0.27	0.00 (0 - 1.1)	0.04	1	Intellectual developmental disorder, X-linked 93	Non-specific
EZHIP	Hearing Loss	0.00	0.27	-0.27	0.00 (0 - 1.1)	0.04	1		Testis, placenta
NUDT10	Hearing Loss	0.00	0.30	-0.30	0.00 (0 - 0.9)	0.02	1		Testis, ovary, brain
SLC7A3	Hearing Loss	0.57	0.88	-0.31	0.19 (0.03 - 1.0)	0.05	1		Thymus

*Only monosomy X women with reported use/offer of hearing aids were included in this analysis. Gene level data are shown - where unique individuals having a variant in a gene are counted and not the number of alleles. A positive effect size for a condition denotes a potential “risk” allele, whereas a negative effect size denotes a potential “protective” allele. Numbers in the hearing loss group are hearing loss n=14, no hearing loss n=33.

Supplementary Table 6. Proportion of X chromosome variants in 45,X women with hearing loss* against the proportion of variants in the same gene in those women without hearing loss.

Gene	Position and Change	Condition	Alt. allele with condition	Alt. allele without condition	gnomAD (v3.1.2) VAF (46,XY)	Effect Size	Odds ratio (95% CI)	Fisher's Exact test (p value)	Bonferroni corrected (p.adj)	HGVSp	Variant effect	PolyPhen	SIFT	CADD	Other in silico predictions (REVEL; SpliceAI)
ARSD	X:2917996 G-C	Hearing Loss	0.86	0.36	0.4617	0.5	9.96 (1.8 - 106)	<0.01	1	p.S224C	missense	NA	NA	8.54	REVEL: 0.252; SpliceAI: 0.00
SLC16A2	X:74421734 T-C	Hearing Loss	0.86	0.42	0.5286	0.44	7.79 (1.4 - 82.9)	0.01	1	p.S33P	missense	benign	tolerated	10.8	REVEL: 0.0850; SpliceAI: 0.00
GAGE12J	X:49323276 G-A	Hearing Loss	0.79	0.36	0.3804	0.43	6.15 (1.3 - 41.2)	0.01	1	p.R28Q	missense	benign	tolerated	4.57	REVEL: 0.0750; SpliceAI: 0.00
GAGE12J	X:49323240 C-G	Hearing Loss	0.79	0.36	0.3939	0.43	6.15 (1.3 - 41.2)	0.01	1	p.P16R	missense	benign	tolerated	0.169	REVEL: 0.0220; SpliceAI: 0.00
GAGE12J	X:49323232 A-T	Hearing Loss	0.79	0.36	0.3519	0.43	6.15 (1.3 - 41.2)	0.01	1	p.R13S	missense	benign	tolerated	0.315	REVEL: 0.0170; SpliceAI: 0.00
GAGE12J	X:49323219 A-G	Hearing Loss	0.79	0.36	0.3476	0.43	6.15 (1.3 - 41.2)	0.01	1	p.Y9C	missense	Probably damaging	tolerated	11.9	REVEL: 0.121; SpliceAI: 0.00
IL1RAPL1	X:28789325 G-A	Hearing Loss	0.86	0.48	0.6269	0.38	6.14 (1.1 - 65.1)	0.02	1	NA	5' UTR	NA	NA	14.1	SpliceAI: 0.0300 (acceptor gain)
CFP	X:47624401 G-A	Hearing Loss	0.5	0.12	0.2208	0.38	6.87 (1.3 - 42.2)	0.01	1	p.N428N	Synony-mous	NA	NA	1.11	SpliceAI: 0.0100 (acceptor gain)

Supplementary Table 6 - continued

Gene	Position and Change	Condition	Alt. allele with condition	Alt. allele without condition	gnomAD (v3.1.2) VAF (46,XY)	Effect Size	Odds ratio (95% CI)	Fisher's Exact test (p value)	Bonferroni corrected (p.adj)	HGVSp	Variant effect	Poly-Phen	SIFT	CADD	Other in silico predictions (REVEL; SpliceAI)
ARSD	X:2914701 G-C	Hearing Loss	0.79	0.42	0.6725	0.37	4.81 (1.0 - 31.9)	0.03	1	p.T358R	missense	NA	NA	0.233	REVEL: 0.00900 SpliceAI: 0.00
VCX	X:7843604 C-G	Hearing Loss	0.5	0.85	0.1768	-0.35	0.19 (0.03 - 0.9)	0.02	1	p.A70G	missense	benign	tolerated	1.85	REVEL: 0.0190; SpliceAI: 0.00
DGKK	X:50403572 C-T	Hearing Loss	0.07	0.42	0.4456	-0.35	0.11 (0.002 - 0.9)	0.02	1	p.L368L	Synony-mous	NA	NA	6.82	SpliceAI: 0.00

*Only monosomy X women with reported use/offer of hearing aids were included in this analysis. Variant level data are shown – where number of alleles are counted. A positive effect size for a condition denotes a potential “risk” allele, whereas a negative effect size denotes a potential “protective” allele. Numbers in the groups analyzed are hearing loss n = 14 and those with no hearing loss n= 33. Bonferroni corrections were made for all genes on the X chromosome where variants were identified. Alt., alternative (minor); CADD, Combined Annotation Dependent Depletion; CI, confidence interval; HGVSp, Human Genome Variation Society protein; LC, low confidence; NA, not applicable; REVEL, Rare Exome Variant Ensemble Learner; SIFT, Sorting Intolerant From Tolerant.

Supplementary Table 7. Selected studies investigating genetic variability and phenotypes in girls and women with Turner Syndrome to demonstrate approaches over time.

Study	PMID	TS (n)	Phenotype(s) studied	Variable(s) considered	Main outcome(s)
Parental X chromosome origin effect					
Mathur et al., 1991	1673045	25	Birth weight, height, neck, cardiovascular, renal, thyroid	Parental X origin	No association of parental X origin with features seen
Chu et al., 1994	7853366	63	Multiple	Parental X origin	Correlation between Xm and maternal/child height percentile. Xm associated with cardiovascular anomalies and neck phenotype in extended analysis
Skuse et al., 1997	9192895	80	Cognitive function	Parental X origin	Association between Xp and social cognitive function
Tsezou et al., 1999	10665663	33	Multiple	Parental X origin	No association of parental X origin with major features seen
Hamelin et al., 2006	16757526	54	Growth and response to rhGH treatment; hearing	Parental X origin	Association between Xm and growth response; Xp associated with sensorineural hearing loss.
Sagi et al., 2007	17192299	83	Multiple	Parental X origin	Potential association between Xm and kidney malformations, lower cholesterol, and higher BMI; and between Xp and ocular anomalies
Ko et al., 2010	20148908	33	Multiple	Parental X origin	No associations with major features (stature, body mass index, cardiac, renal, skeletal, lymphatic, hearing or ocular systems), although height associated when maternal X (Xm) inherited
Davernay et al., 2012	22593588	180	Growth and response to rhGH treatment	Parental X origin	No association of parent X origin with growth seen
Alvarez-Nava et al., 2013	23731950	93	Multiple	Parental X origin	Potential association between Xm and adult dyslipidemia; no other effects seen on features, anomalies, or growth
Malhotra et al., 2020	32813677	59	Multiple	Parental X origin	Xm associated with skeletal features (short neck; short 4th metatarsal); no effect of parental X chromosome on biochemical profile or body composition
Common genetic variants influencing phenotype (TS-specific)					
Larizza et al., 1989	2491644	46	Autoimmunity	HLA type	Potential association between HLA-A31 and autoimmunity
Weiss et al., 2007	17164267	93 (+77)	Social cognition	*EFHC2*	Quantitative trait locus SNP (rs7055196) associated with facial fear recognition
Zinn et al., 2008	17948898	97	Social cognition	*EFHC2*	No association with rs7055196 found
Bianco et al., 2010	20696024	142	Autoimmunity	*PTPN22* (C1858T)	Risk allele (T) for autoimmune disease
Villanueva-Ortega et al., 2017	28627089	109	Autoimmunity	*MYO9B* (rs2305767)	Associated with coeliac disease
Dos Santos et al., 2018	30508004	86	Multiple	*CTLA-4* (rs231775)	Associated with obesity
Barrientos-Rios et al., 2019	30887870	61	Multiple	*KL* (rs9536282)	Associated with renal malformations
Common genetic variants influencing phenotype (general)					
Binder et al., 2008	17973940	48	Height after rhGH treatment	*GHR-d3*	Association between d3/d3 and greater response to GH treatment, and lower BMI
Braz et al., 2014	24905066	65	Height after rhGH treatment	*SOCS2* (rs3782415)	Height response to rhGH associated with *SOCS2*, also *GHR-d3* and *IGFBP3* (-202C) (also, Braz et al., 2012)
Stevens et al., 2016	27651465	132	Growth response to rhGH treatment	48 SNPs	Associated with variants in *ESR1* and *PTPN1*
Barrientos-Rios et al., 2019	30887870	61	Multiple	*CYP27B1* (rs4646536)	Associated with low BMD
Scalco et al., 2019	31671406	91	Height, uterine growth, BMD	*ESR1* (rs2234693)	C/C associated with lower femoral and hip BMD
Exonic variants influencing phenotype					
Corbitt et al., 2018	30281655	188	Bicuspid aortic valves, aortic root dimension z-scores	*TIMP3* (rs11547635) *(TIMP1)*	*TIMP3* variants associated with aortopathy. Single copy of *TIMP1* (X chromosome) also associated with aortopathy. Combinatorial effect.
Pinnaro et al., 2023	36929416	208	Bicuspid aortic valves	*CRELD1*	Rare variants in *CRELD1* enriched in TS girls/women with bicuspid aortic valves

This table highlights several key approaches to studying genetic variability and phenotype with TS over the past 35 years. Potential associations between structural variants and phenotypes are not included, nor are studies suggesting an enrichment of genetic variation in TS compared to control populations. Studies with fewer than 25 women with TS were excluded. The data shown here do not represent a systematic review of all positive and negative findings. In many situations, subsequent studies were published that presented contradictory or negative findings. There is likely a reporting bias to studies that generate positive findings, but these are presented here to give an overview of approaches taken. BMD, bone mineral density; BMI, body mass index; GHRd3, exon3 deleted variant of the growth hormone receptor; n, number; rhGH, recombinant human growth hormone; PMID, PubMed identification number; SNP, single nucleotide polymorphism.

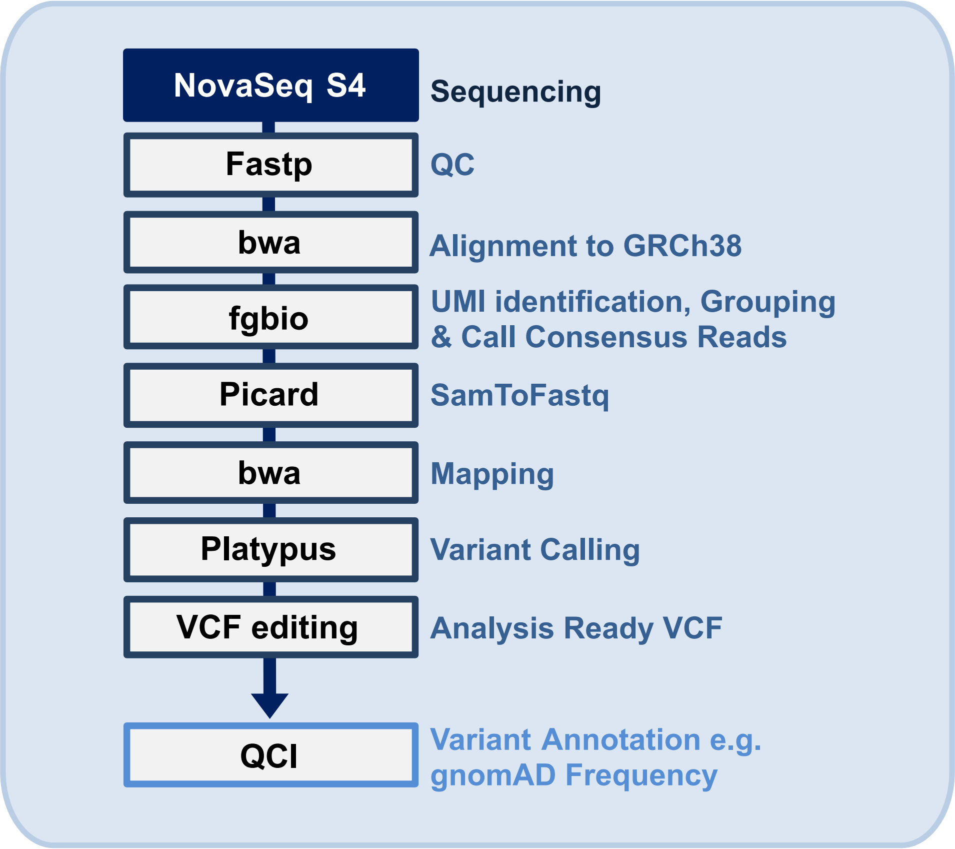

Supplementary Figure 1. Bioinformatic pipeline for Variant Call Format (VCF) file generation and data filtering analysis. Exome data pipeline for genome alignment and variant calling. bwa, Burrows-Wheeler aligner; GRCh38, Genome Reference Consortium Human Build 38; QC, quality control; QCI, Qiagen clinical insight; UMIs, unique molecular identifiers; VCF, variant call format.

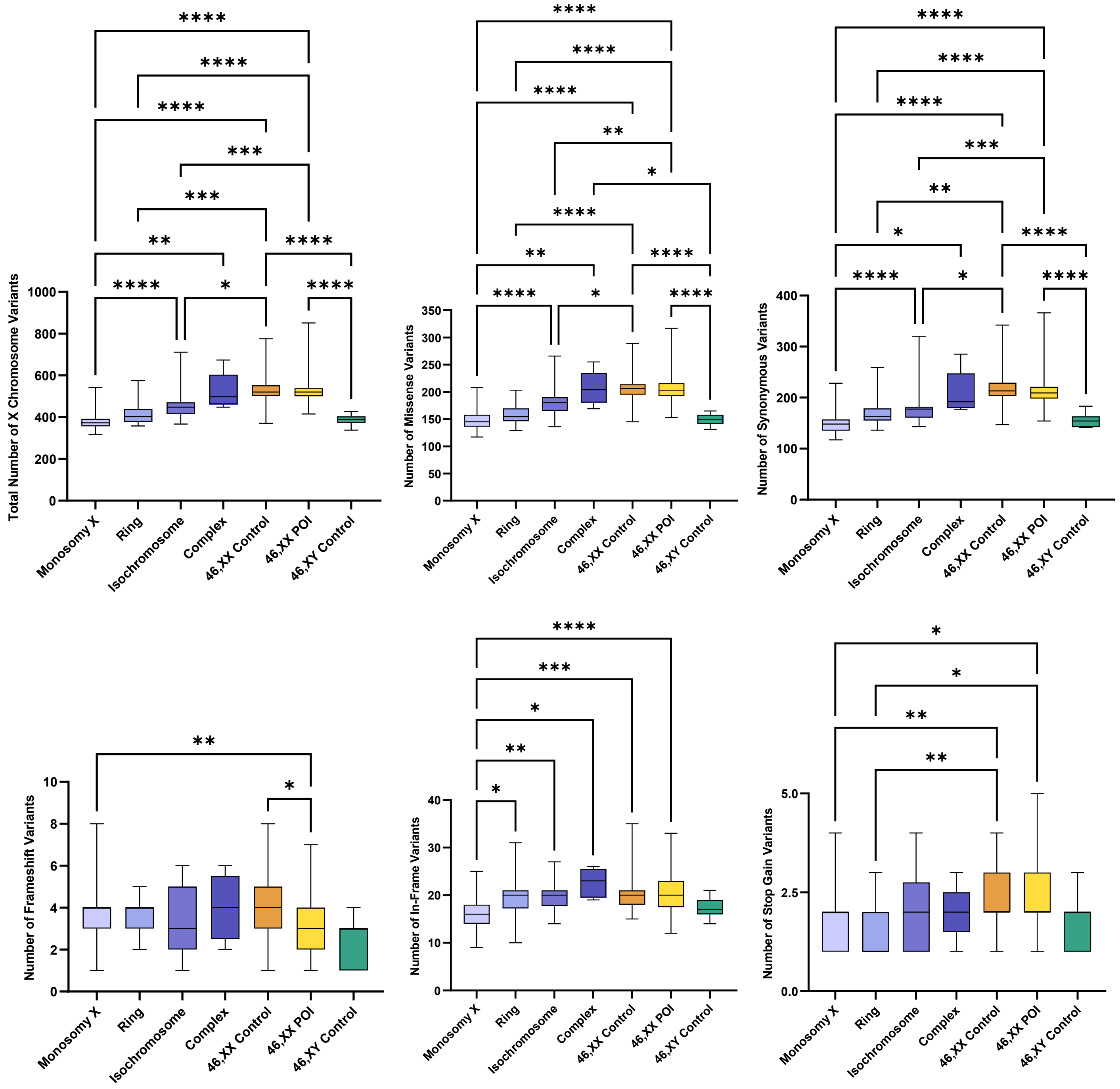

Supplementary Figure 2. Total number of X chromosome variants in each cohort with statistical analysis. Data are represented as box plots showing the lower quartiles, upper quartiles and the median with the whiskers showing the range of the data. The number of X chromosome variants appeared proportionate to the amount of X chromosome material. Statistical differences between groups are shown using Kruskal-Wallis one-way analysis of variance. Statistical significance is shown where: *, P value<0.05; **, P value<0.01; ***, P value<0.001; ****, P value<0.0001. POI, primary ovarian insufficiency.

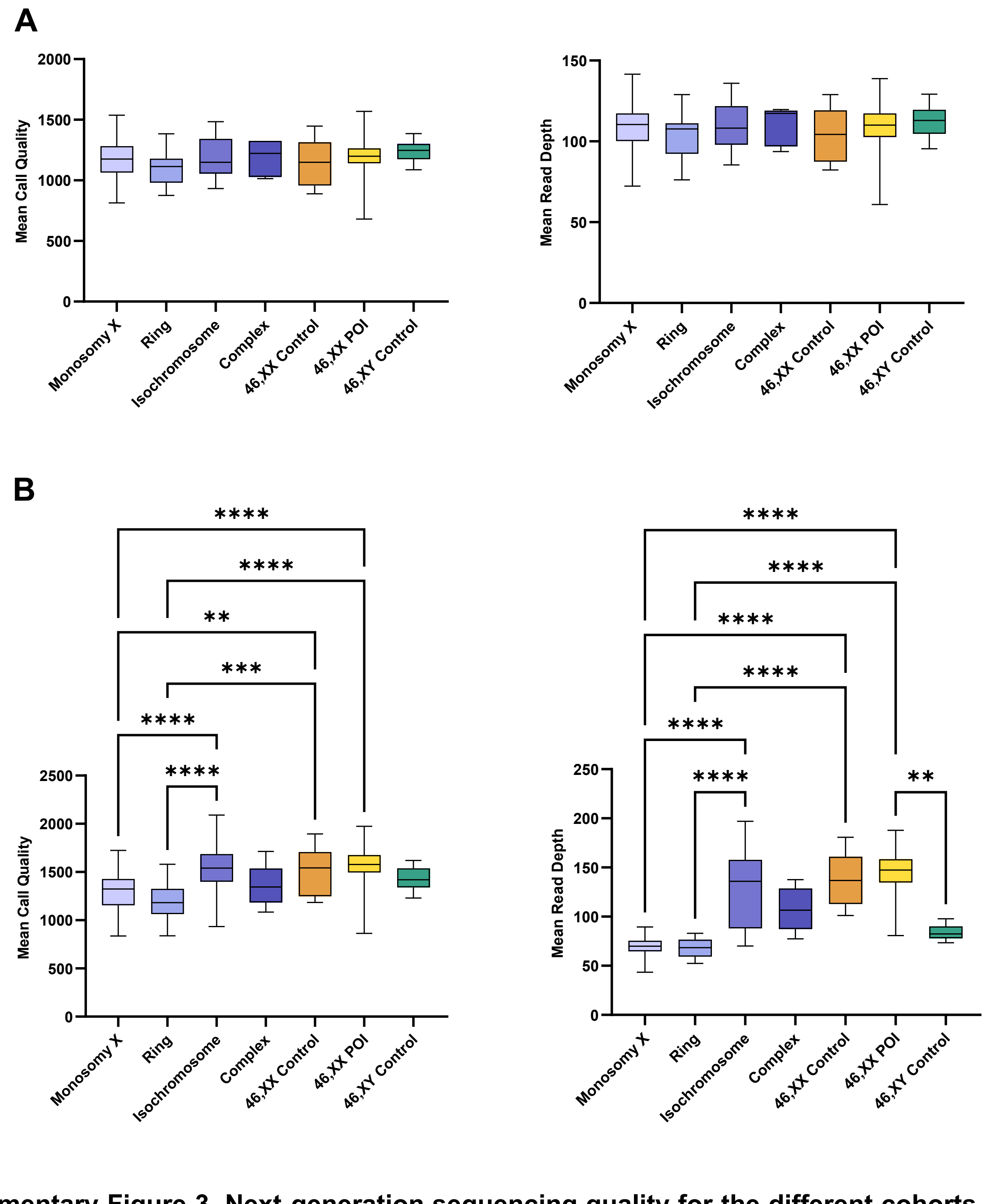

Supplementary Figure 3. Next-generation sequencing quality for the different cohorts studied. (A) Mean call quality and read depths for the autosomes. No statistically significant differences (P value <0.05) were found between any of the cohorts (Kruskal-Wallis one-way analysis of variance). **(B)** Mean call quality and mean read depth for the X chromosome. Data are represented as box plots showing the lower quartiles, upper quartiles, and the median (of mean values) with the whiskers showing the range of the data. Statistical significance is shown where: **, P value<0.01; ***, P value<0.001; ****, P value<0.0001. POI, primary ovarian insufficiency.

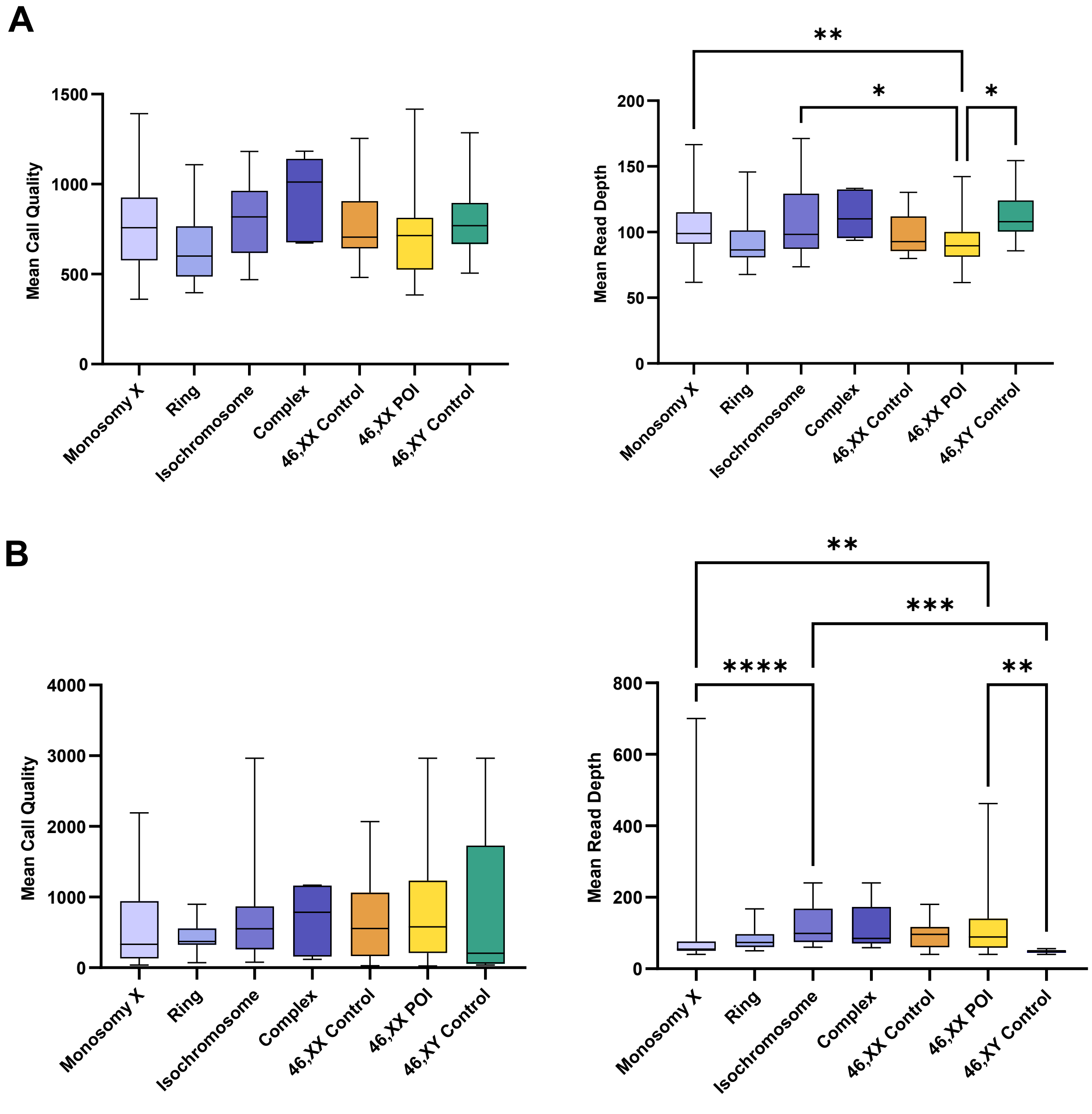

Supplementary Figure 4. Next-generation sequencing quality for the different somatic filtered variants in the cohorts studied. (A) Mean call quality and read depths for the somatic filtered autosomes. **(B)** Mean call quality and mean read depth for somatic filtered X chromosome. Data are represented as box plots showing the lower quartiles, upper quartiles, and the median (of mean values) with the whiskers showing the range of the data. Statistical differences between groups are shown using Kruskal-Wallis one-way analysis of variance. Statistical significance is shown where: *, P value<0.05; **, P value<0.01; ***, P value<0.001; ****, P value<0.0001. POI, primary ovarian insufficiency.

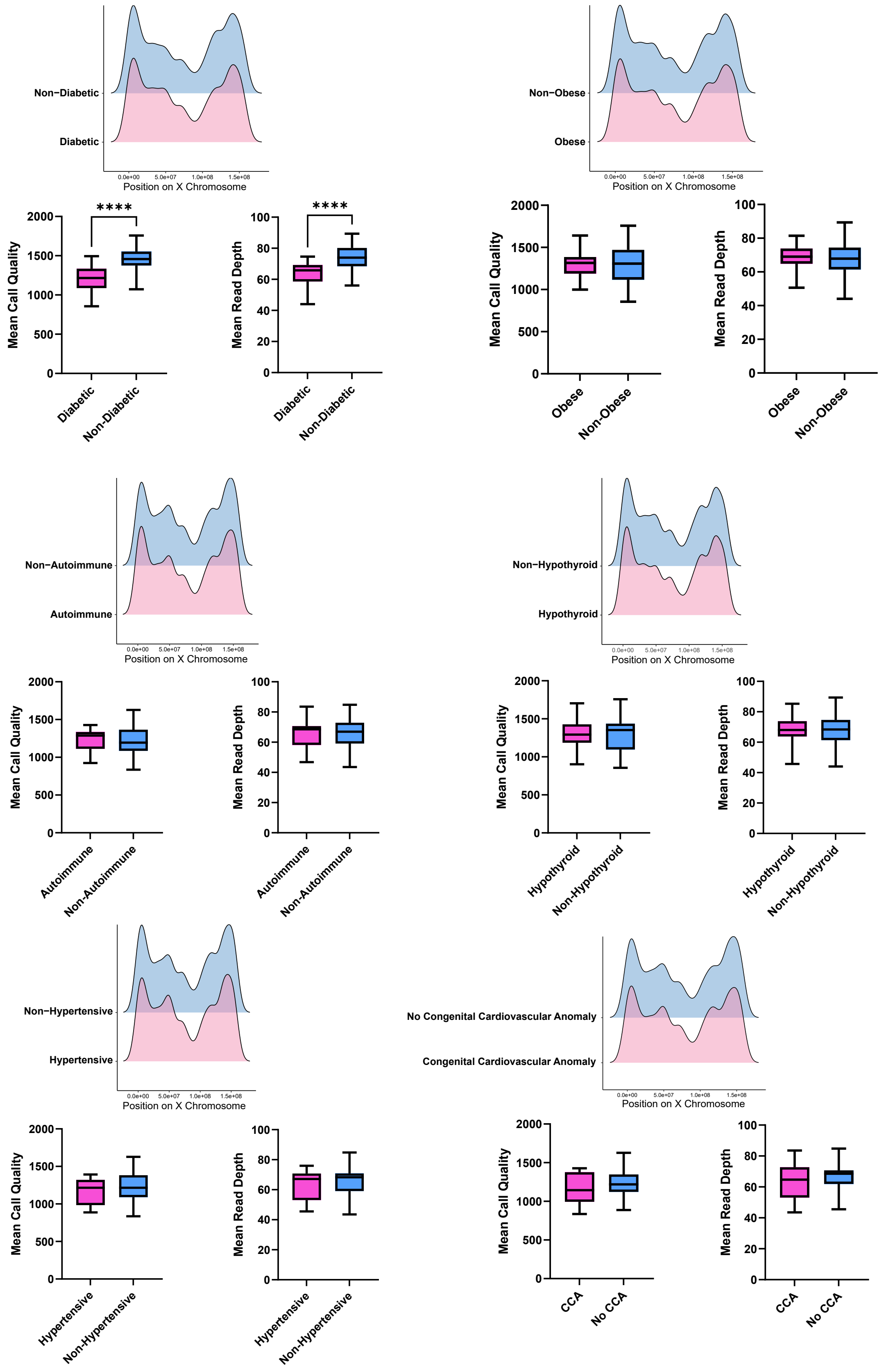

Supplementary Figure 5. Mean call quality and mean read depth of condition data sets included in the X chromosome gene variant enrichment analysis related to phenotype. Data are represented as ridgeplots (density of sequencing across the X chromosome) and box plots showing the lower quartiles, upper quartiles, and the median (of mean values) with the whiskers showing the range of the data. Data for group with a condition are shown in pink and those without a condition are shown in blue. Statistical differences between groups are shown using non-parametric T-tests (Mann Whitney) ****, P value<0.0001. CCA, Congenital cardiovascular anomaly.

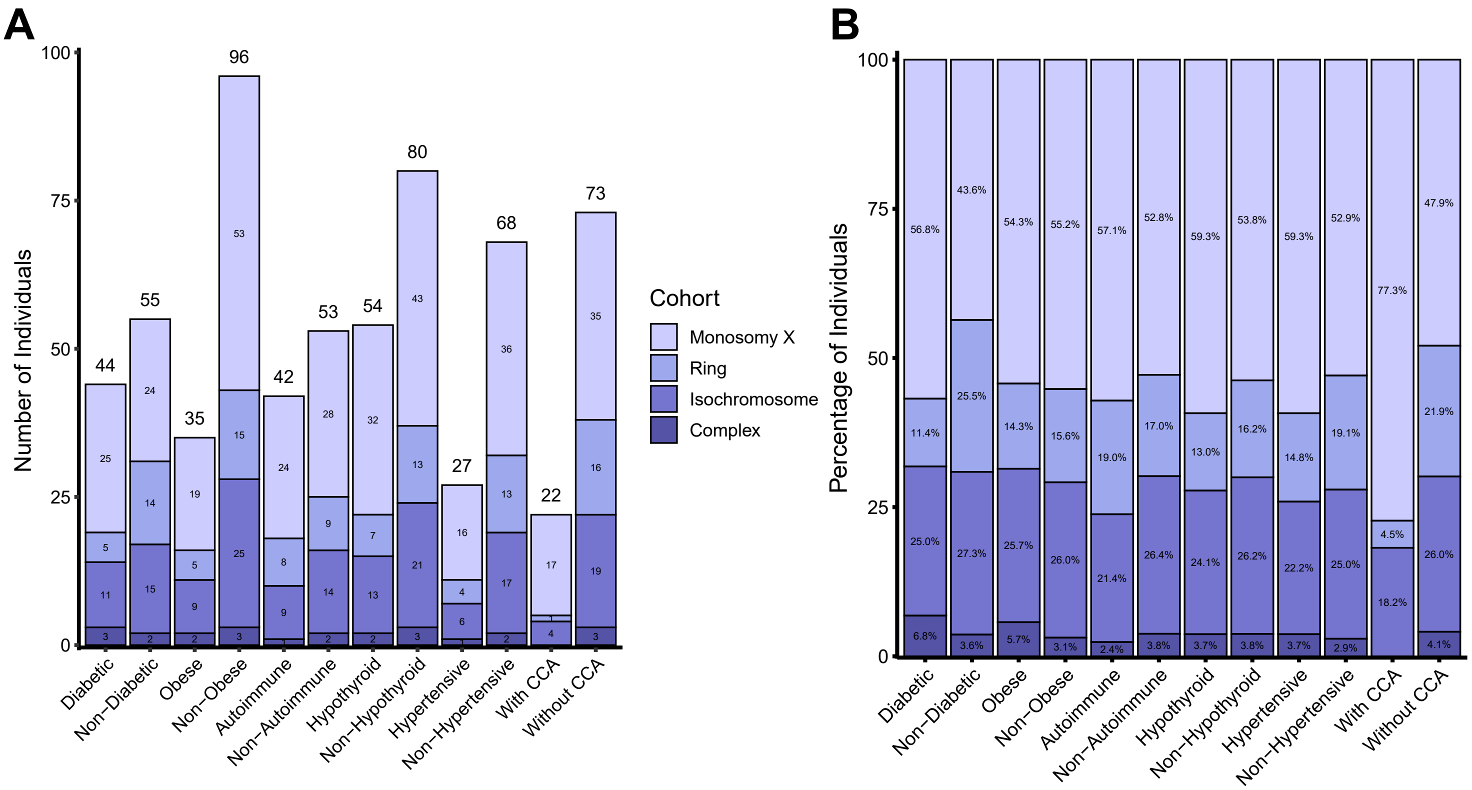

Supplementary Figure 6. Turner syndrome cohort number and as percentage with conditions and those without used as controls for comorbidity analysis. (A) Total group numbers are labeled on top, with karyotype numbers underneath and colored according to their respective karyotypes. **(B)** Karyotype percentages for each group are shown and colored according to their respective karyotypes. Note: Women with CCA had a higher proportion with monosomy X compared to ring mosaicism (Chi-Sq p-value 0.03) consistent with the hypothesis that a single copy of TIMP1 is a driver for cardiac anomalies (Corbitt et al, 2018).

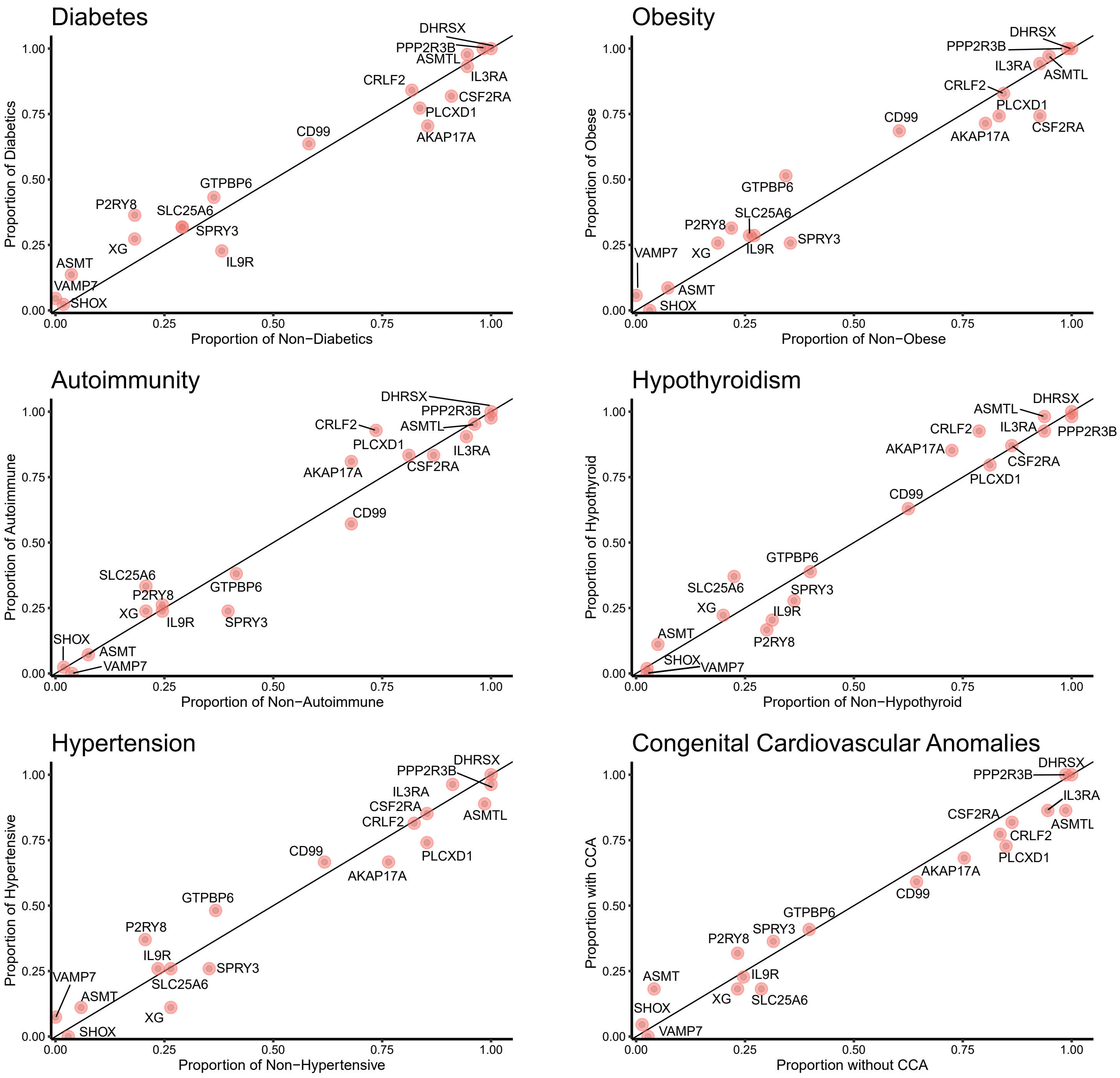

Supplementary Figure 7. Scatterplots (gene level) of the proportion of PAR genes in unique individuals with Turner syndrome with a condition against the proportion of variants in the same gene in unique individuals of Turner women without the condition. The number of data points at any given coordinate is shown by the intensity of the circle.

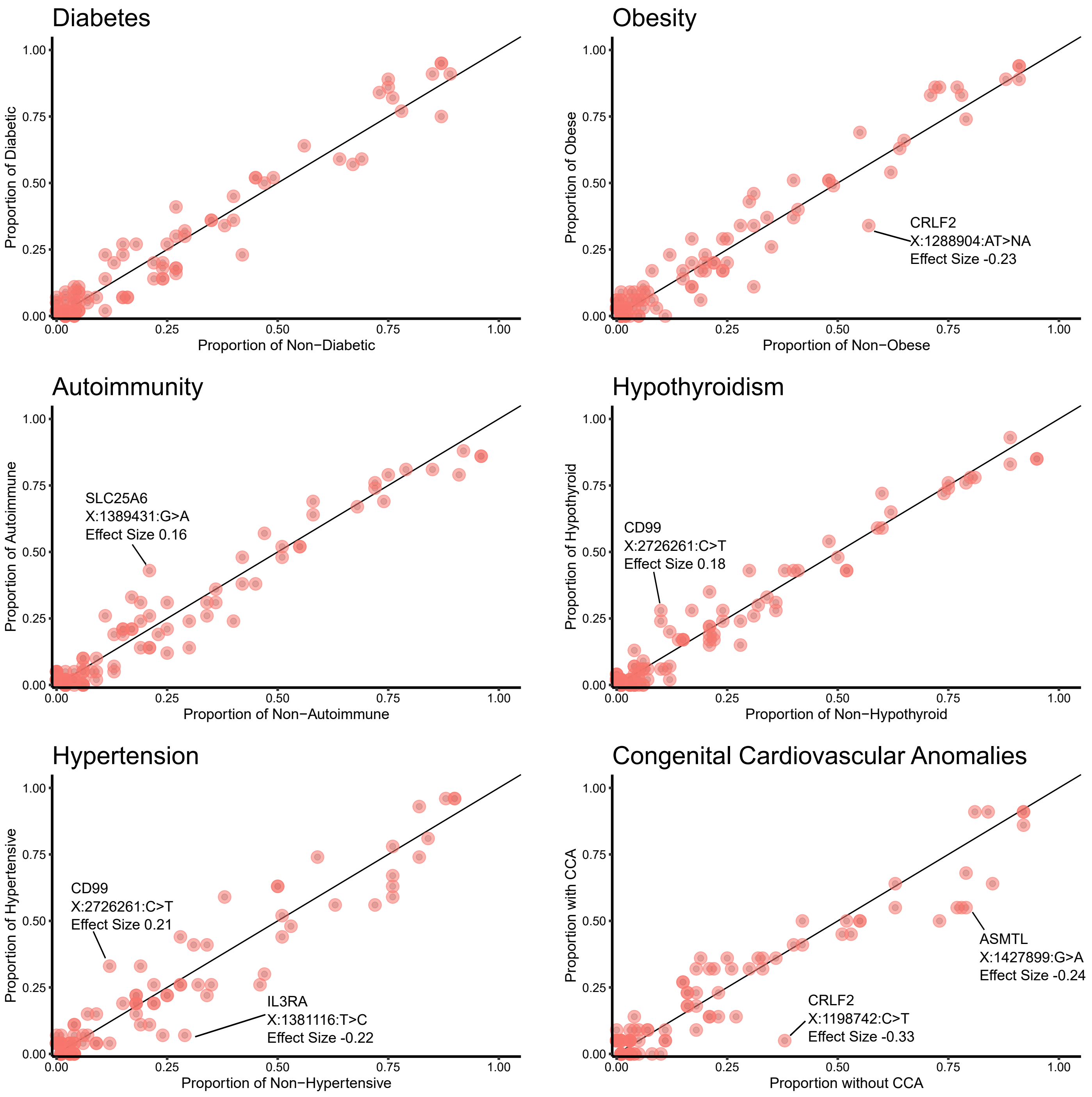

Supplementary Figure 8. Scatterplots (variant level) of the proportion of PAR gene variants in Turner syndrome women with a condition against the proportion of variants in the same gene in those women without the condition. The number of data points at any given coordinate is shown by the intensity of the circle. Selected genes that have potential “risk” and “protective” effects are labeled.

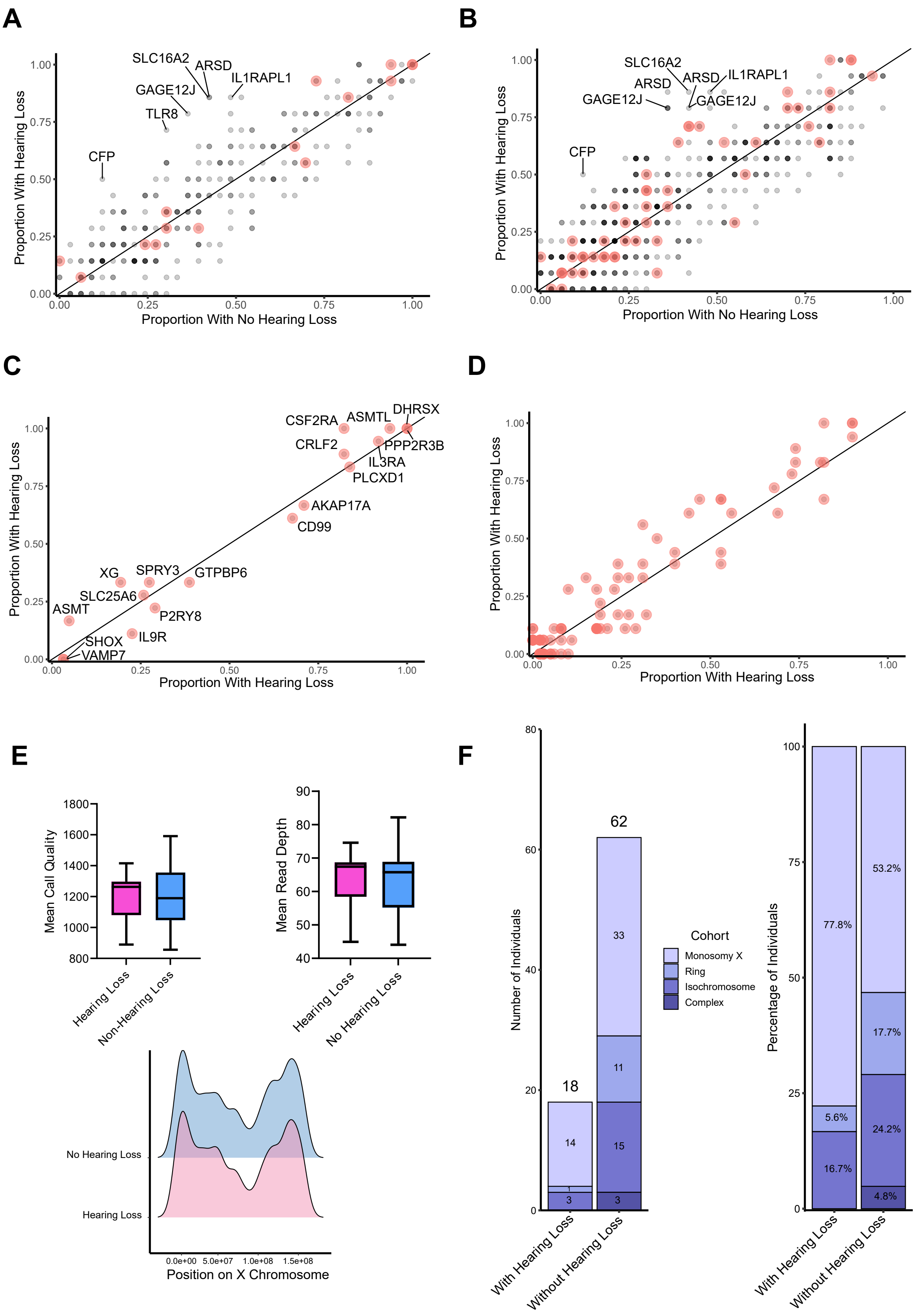

Supplementary Figure 9. Hearing loss* data in monosomy X and extended PAR gene analysis in women with Turner Syndrome. *Only Turner syndrome women with reported use/offer of hearing aids were included in this analysis. **(A)** Scatter plots (gene level) and **(B)** scatter plots (variant level) of the proportion of X chromosome gene or variants in 45,X women with hearing loss against the proportion of variants in the same gene or variant in those women without hearing loss. **(C)** Scatter plots (gene level) and **(D)** scatter plots (variant level) of the proportion of PAR gene variants in women with Turner syndrome with hearing loss against the proportion of variants in the same PAR gene or variant in those women without hearing loss. **(E)** Mean call quality and mean read depth of hearing groups (see Supplementary Figure 5). **(F)** TS cohort overview.
